# Dynamic gene expression changes in response to micronutrient, macronutrient, and multiple stress exposures in soybean

**DOI:** 10.1007/s10142-019-00709-9

**Published:** 2019-10-26

**Authors:** Jamie A. O’Rourke, Chantal E. McCabe, Michelle A. Graham

**Affiliations:** 1 Corn Insects and Crop Genetics Research Unit, USDA-ARS, Ames, IA 50011 USA; 2 Department of Agronomy, Iowa State University, 1567 Agronomy Hall, Ames, IA 50011 USA

**Keywords:** Iron, Phosphate, RNA-seq, Gene expression, Nutrient stress

## Abstract

Preserving crop yield is critical for US soybean production and the global economy. Crop species have been selected for increased yield for thousands of years with individual lines selected for improved performance in unique environments, constraints not experienced by model species such as *Arabidopsis*. This selection likely resulted in novel stress adaptations, unique to crop species. Given that iron deficiency is a perennial problem in the soybean growing regions of the USA and phosphate deficiency looms as a limitation to global agricultural production, nutrient stress studies in crop species are critically important. In this study, we directly compared whole-genome expression responses of leaves and roots to iron (Fe) and phosphate (P_i_) deficiency, representing a micronutrient and macronutrient, respectively. Conducting experiments side by side, we observed soybean responds to both nutrient deficiencies within 24 h. While soybean responds largely to –Fe deficiency, it responds strongly to P_i_ resupply. Though the timing of the responses was different, both nutrient stress signals used the same molecular pathways. Our study is the first to demonstrate the speed and diversity of the soybean stress response to multiple nutrient deficiencies. We also designed the study to examine gene expression changes in response to multiple stress events. We identified 865 and 3375 genes that either altered their direction of expression after a second stress exposure or were only differentially expressed after a second stress event. Understanding the molecular underpinnings of these responses in crop species could have major implications for improving stress tolerance and preserving yield.

**Electronic supplementary material:**

The online version of this article (10.1007/s10142-019-00709-9) contains supplementary material, which is available to authorized users.

## Introduction

Iron (Fe) is an essential micronutrient for plants, involved in multiple physiological processes including photosynthesis and electron transport. Though abundant, environmental conditions including high pH, calcareous soil composition, and aerobic conditions often render Fe insoluble and unavailable for plant use (Marschner 1995). Approximately 30% of cultivated soils worldwide are calcareous, including those in the Upper Midwestern United States where over 90% of US soybeans are produced. Conversely, excessive Fe is toxic, resulting in plant death. To regulate iron uptake, plants have evolved two strategies. Soybeans utilize the strategy I response, which involves increased ferric reductase activity at the root surface to convert ferric (Fe^3+^) to ferrous (Fe^2+^) iron, which can then be transported into the plant root by specific Fe transporters. In *Arabidopsis thaliana*, genes regulating these responses have largely been identified through reverse genetic approaches (Henriques et al. 2002; Rogers and Guerinot 2002; Vert et al. 2002; Bauer et al. 2007; Long et al. 2010; Yan et al. 2016). In contrast, soybean studies over the last 35 years have used traditional quantitative trait locus (QTL) mapping in field conditions, sequenced-based introgression mapping, and gene expression studies to investigate Fe deficiency tolerance (Lin et al. 1997, 2000; O’Rourke et al. 2009; Severin et al. 2010; Peiffer et al. 2012; Atwood et al. 2014; Moran Lauter et al. 2014). These studies have found the soybean response to –Fe (iron deficiency stress) includes increased iron uptake/transport, DNA replication/methylation, and defense. Similar responses have not been identified in model species.

Phosphorous, in its orthophosphate form (P_i_), is one of the most rate-limiting macronutrients in agricultural production. Much like iron, phosphate is often plentiful in the soils, but slow diffusion and high fixation within the soil leave little P_i_ available for plant utility (Shen et al. 2011). P_i_ is commonly applied as a fertilizer, but plants only utilize 20% of the applied fertilizer, and the rest is lost through run-off (Cordell et al. 2009). This contributes to environmental problems such as eutrophication of aquatic systems resulting in anoxic conditions detrimental to vertebrate and insect populations and enhanced algal blooms (Carpenter 2008; Schindler et al. 2008; Cordell et al. 2009). Mined rock phosphate is a finite resource that will become more expensive as easily accessible reserves are mined to depletion within the next 50 years. Thus, understanding how plants acquire and utilize P_i_, and how low P_i_–tolerant plants thrive in harsh growing conditions, is critical to improving agricultural systems. In soybean, studies have characterized the expression pattern and role of genes important in model species P_i_ homeostasis (Liao et al. 2003; Qin et al. 2012; Fan et al. 2013; Song et al. 2013; Li et al. 2014; Yao et al. 2014; Zhang et al. 2014, Zhang et al. 2016a, b). Classical QTL studies have identified P_i_ efficiency QTL in soybean (Liang et al. 2010; Zhang et al. 2009, 2014, 2017), one of which included RNA-seq analysis of two recombinant inbred lines (RILs) with contrasting tolerance to P_i_ deficiency stress (–P_i_). Given the paucity of gene expression data for –P_i_-stressed soybean, the data provided by our study provides a vital resource to investigate the genetic responses and molecular pathways involved in soybean’s –P_i_ response in a US milestone cultivar. While researchers investigating nutrient deficiency have long noted the similarity between –Fe and –P_i_ responses, this is the first study to directly examine whether the same genes and pathways are responding similarly to both stresses.

This study analyzes and compares soybean responses to –Fe and –P_i_ deficiencies. In addition, this study includes multiple timepoints allowing us to investigate gene expression changes in response to initial stress, recovery from stress, and a second stress exposure. These findings will help improve our understanding of soybean’s response to abiotic stress, a critical component to protecting soybean yield.

## Materials and methods

### Experimental design

**Fig. 1:**
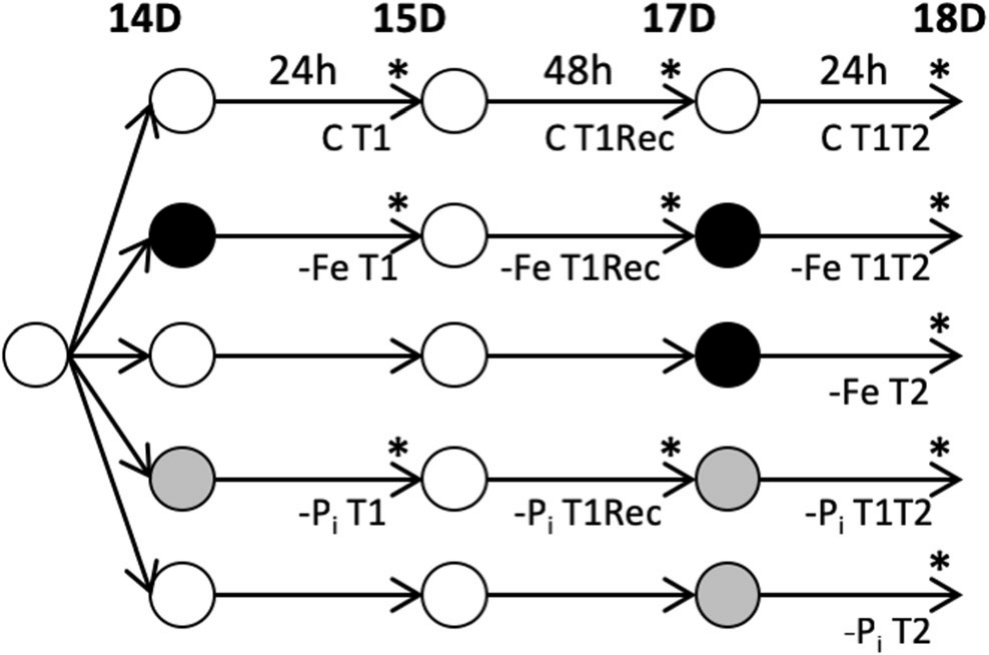
Experimental design to examine repeated short-term nutrient deficiency stress on gene expression. After 7 days on germination paper, plants were moved to optimal (white) nutrient solution for 7 days. On day 14, plants were moved to one of three new nutrient solutions: optimal (white), –Fe (black), or –P_i_ (gray) for 24 h at which time, roots and leaves of a subset of plants were collected separately (T1). Sample times are indicated with an asterisk (*). All remaining plants were moved to new optimal solutions for 48 h at which time, samples from T1Rec plants were collected. After T1Rec collection, all remaining plants were again moved to new solutions for 24 h at which time, tissue from all remaining plants was collected. In total, there were five groups of plants: group 1, control (C); group 2, –FeT1, –FeT1Rec, and –FeT1T2; group 3, –FeT2; group 4, –P_i_T1, –P_i_T1Rec, and –P_i_T1T2; and group 5, –P_i_T2. These groups yielded 22 unique samples, 11 from roots and 11 from leaves. Sample names (minus the tissue designation) are listed under the harvest timepoint and are as follows: CT1, CT1Rec, CT1T2, –FeT1, –FeT1Rec, –FeT1T2, –FeT2, –P_i_T1, –P_i_T1Rec, –P_i_T1T2, and –P_i_T2

To investigate responses to repeated iron and phosphate stress required eight different treatments: early stress (–FeT1 and –P_i_T1), recovery (–FeT1Rec and –P_i_T1Rec), repeated stress (–FeT1T2 and –P_i_T1T2), late stress (–FeT2 and –P_i_T2), and non-stress controls collected at each timepoint (Fig. 1). All plants were grown simultaneously to facilitate direct comparisons between treatments. Since no P_i_ deficiency studies have been performed on US soybean lines, we used Clark, an iron-efficient soybean line (Bernard et al. 1991) commonly used in our research program and which also responds to –P_i_ conditions (Online Resource 1). Clark seeds were started on germination paper for 7 days and then transferred to optimized hydroponic solutions as described previously (Chaney et al. 1992). Cotyledons were removed at the time of transfer. Supplemental nutrient solutions were added daily. All plants were grown in hydroponics for 7 days under full nutrient conditions. At 14 days after sowing on germination paper, one treatment set was moved to –Fe (50 μM Fe(NO_3_)_3_), one treatment set was moved to –P_i_ (0 μM P_i_), and the remaining sets were moved to new full nutrient solutions. Plants remained in these solutions for 24 h at which time, a subset of plants from each treatment and controls was harvested (–P_i_T1, –FeT1, and ControlT1). All remaining plants were then moved to new optimal nutrient solutions for recovery. After 48 h in full nutrient solutions, a subset of plants from each initial treatment and controls was harvested (–P_i_T1Rec, –FeT1Rec, ControlT1Rec). All remaining plants were then moved into new solutions for 24 h. One treatment set was returned to –Fe (50 μM Fe(NO_3_)_3_, –FeT1T2), one treatment set was returned to –P_i_ (0 μM P_i_, –P_i_T1T2), one treatment set (previously unstressed) was moved to –Fe (50 μM Fe(NO_3_)_3_) for late –Fe stress exposure (–FeT2), one treatment set (previously unstressed) was moved to –P_i_ (0 μM P_i_) for late –P_i_ stress exposure (–P_i_T2), and the final set was moved to fresh nutrient sufficient conditions (ControlT2).

### Phenotyping

**Fig. 2:**
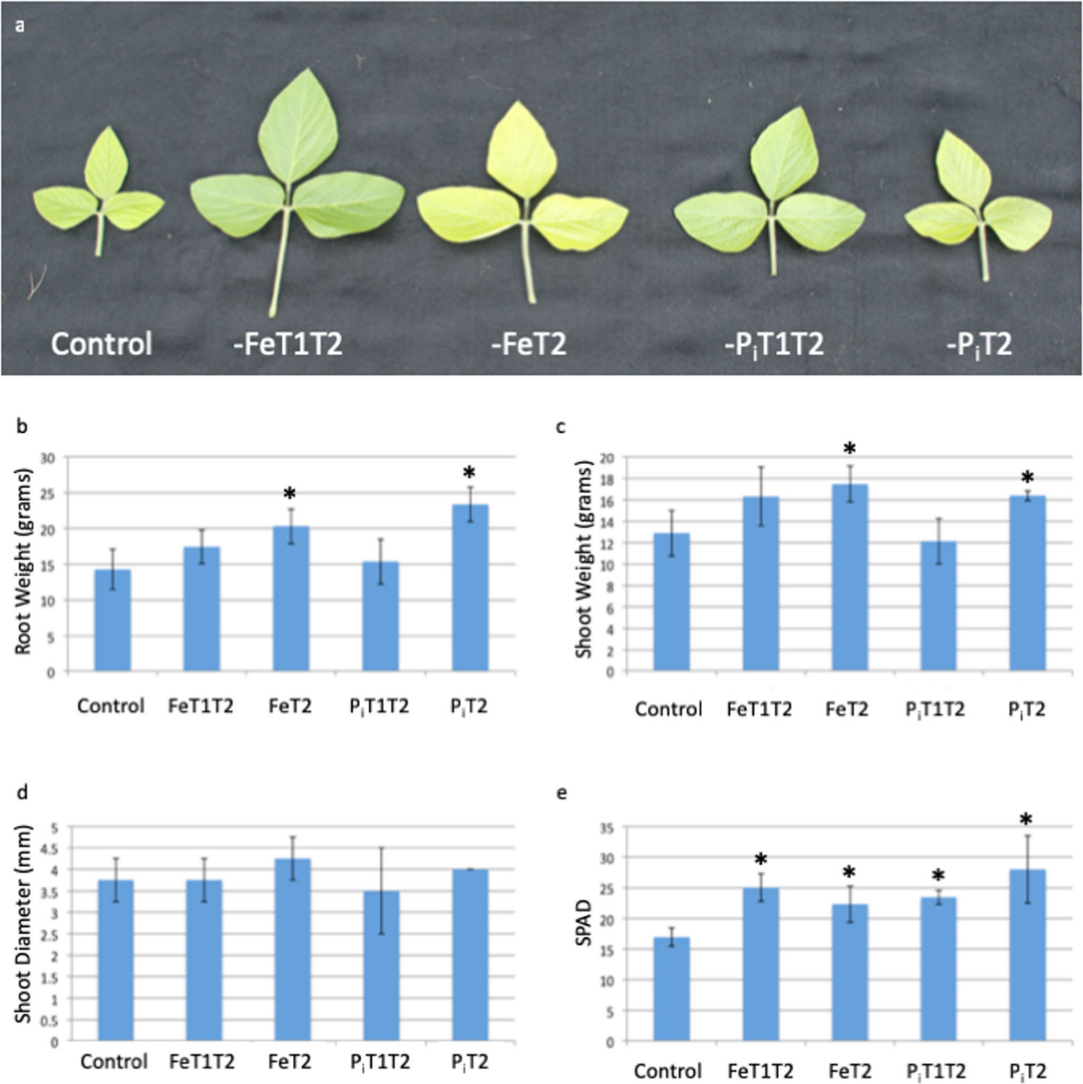
Phenotypic effect of short-term nutrient deficiency stress. Plants were grown as described in Fig. 1 except all plants were retained until the end of the study for phenotyping. **a** Sixth trifoliate. The average size of trifoliates from stressed plants is larger than that of plants maintained in optimal nutrient solutions throughout the experiment. **b** Root weight (grams). **c** Shoot weight (grams). Nutrient deficiency stress at T2 results in statistically significant increases in root (**b**) and shoot (**c**) weights. **d** Shoot diameter (mm), not significantly affected by nutrient deficiency stresses. **e** Chlorophyll content, as measured by SPAD, confirms plants exposed to multiple rounds of nutrient deficiency (both –Fe and –Pi) and late (T2) nutrient deficiency contain enhanced chlorophyll content compared to control plants

Given the large number of plants needed, the experiment was completed in two phases. In phase 1, the growth conditions described above were used to gather phenotypic data in response to early, repeated, and late stress exposures. SPAD readings on all present trifoliates and shoot height measurements were started 3 days after T2 stress collection timepoint and repeated daily for 6 days. Plants were harvested on day 7 (18 days in hydroponics), and additional phenotypic data including plant height, shoot weight, root length, root weight, and shoot diameter were collected (Fig. 2).

### RNA extraction and sequencing

In phase 2, we repeated the experiment and collected the fourth trifoliate and the entire root system of each treatment set for RNA-seq analyses. Four biological replicates, each a single plant, were collected and immediately frozen in liquid nitrogen. RNA was extracted using RNeasy kits (Qiagen, Valencia, CA). Contaminating DNA was removed using the Ambion TURBO DNA-free kit (Ambion, Austin, TX). RNA was purified and concentrated using the Qiagen RNeasy MinElute Cleanup kit (Qiagen, Germantown, MD). Sample purity and quantification were measured on the NanoDrop ND-1000 spectrophotometer (Thermo Fisher Scientific, Waltham, MA) and QIAxcel (Qiagen, Germantown, MD) with concentrations over 500 ng/μL and RIS scores > 7. RNA from three biological replicates was submitted to the Iowa State DNA Facility. Library preparation was performed from 4 μg of total RNA using the Illumina® TruSeq RNA library preparation kit (v2), according to the manufacturer’s directions. Subsequent 100-bp single end sequencing was performed using the Illumina HiSeq 2500 (Illumina, San Diego, CA). All reads have been submitted to the National Center for Biotechnology Information (http://www.ncbi.nih.gov/sra) under the BioProject accession PRJNA544698.

### Quality control and read mapping

Read quality was accessed by FastQC (Andrews 2010). Reads with quality scores greater than 20 and longer than 30 bases were mapped to the soybean genome (Glyma.Wm82.a2.v1, (Gmax2.0), https://phytozome.jgi.doe.gov/pz/portal.html#!info?alias=Org_Gmax) using Tophat2 (version 2.1.1) (Langmead et al. 2009) with default parameters except a maximum intron length of 10,000 bp. The program SAMtools (version 1.3.1) (Li et al. 2009) was used to retain uniquely mapping reads. Sample data was imported into RStudio (version 0.98.945) (Team RStudio 2015) for further analysis. Leaf and root samples were normalized independently using DESeq (version 1.14.0) (Anders and Huber 2012). Biological replicates were analyzed to ensure the expression between replicates was consistent. These analyses included a multiple dimensional scaling (MSD) plots of all leaf samples and all root samples. The MDS analyses demonstrated clustering of all biological replicates and distinguished treatment differences. Additionally, MA plots show pairwise comparisons of samples within tissue and within timepoints which were made using the scatmat function of the graphics program ggplot2 (version 0.9.3.1) (Wickham 2016) to visualize the consistency between replicates. The sum of these analyses determined the expression profile of a single root sample (ControlT1 recovery) and four leaf samples (ControlT1 recovery, ControlT2, ControlT1T2, and –FeT2) was statistically different from that of the remaining biological replicates. These samples were removed from the analyses and the data renormalized. The statistical tests were repeated, and all biological replicates were found to be statistically similar (Online Resources 2, 3, and 4).

### Identification of differentially expressed genes

Using the renormalized data, edgeR (Robinson et al. 2010) analyses identified differentially expressed genes (DEGs). Differential expression analyses compared plants exposed to nutrient stress to plants grown continuously in full nutrient conditions at the same timepoint. DEGs were considered significant if their fold change was > 2, the *P* value was < 0.05, and the FDR was < 0.05. Gene expression profiles for all DEGs identified in leaves and roots are available in Online Resources 5 and 6, respectively. Expression profiles of DEGs were visualized using either raw fold change data and manual clustering in Treeview (Saldanha 2004) or *Z* scores and hierarchical clustering in ggplot2 (Wickham 2016).

### Semi-quantitative real-time PCR analysis

To further validate the differential expression identified by RNA-seq, sixteen genes were selected for qRT-PCR analysis. The genes include transcription factors (TFs), candidate genes underlying QTLs, and those with annotations which indicate they could play a major role in the soybean nutrient response. ELF1b was used as a housekeeping gene as identified and utilized by Yuan et al. (2016a). Gene-specific primers were designed using the PrimerQuest tool at idtdna.com with default parameters except primer *T*_m_ = 60 °C and primer size = minimum of 14 and optimum of 16, and 2 3′ GC clamps were requested. Real-time PCR was conducted using 50 ng of RNA as a template and the SuperScript® III Platinum® SYBR® Green One-Step qRT-PCR kit from Illumina. PCRs were run on two biological replicates, each with two technical replicates, as 25 μL reactions (0.5 μL SuperScript III, 12.5 μL 2× SYBR® Green reaction mix, 0.5 μL F primer (10 μM), 0.5 μL R primer (10 μM), 0.5 μL ROX Reference dye, 50 ng RNA, and water up to 25 μL). PCRs were run in a Stratagene® Mx3000P with a 3-min cDNA synthesis step at 50 °C and a 5-min denaturation step at 95 °C. Amplification parameters were 40 cycles of 95 °C for 15 s, 53 °C for 30 s, and 72 °C for 1 min. This was followed by a dissociation curve analysis to confirm the reaction specificity.

### Gene annotations

DEGs were assigned functional annotations using the annotation tool on SoyBase (www.soybase.org/genomeannotation/). This tool assigns Gene Ontology terms to each soybean gene using the Gene Ontology (GO) of the best *Arabidopsis thaliana* homolog as identified by BLASTP (*E* < 10^−6^). TFs were identified using the SoyDB transcription factor database published by Wang et al. (2010). To identify significantly overrepresented (corrected *P* < 0.05) GO terms or transcription factor families (TFFs) within a dataset relative to the soybean genome, a Fisher’s exact test (Fisher 1966) with a Bonferroni correction (Bonferroni 1935) was used (Morales et al. 2013). To reduce the number of terms, GO terms with completely overlapping gene lists were assigned to the highest-order significantly overrepresented GO term. Overrepresented GO terms were used to assign biological function and classification to heatmap clusters.

### Correlation with QTLs

To correlate gene expression with previously identified QTL regions, we queried the SNP markers from each of the previous Fe and P_i_ QTL studies against the SoyBase genome browser (www.soybase.org). Genomic regions between SNP markers were assigned as the QTL region. Genes located within the region were identified using the Williams 82 a2.v1 gene position file available at www.phytozome.net. Genes differentially expressed at each timepoint were identified from our datasets. Sequences of genes within the Fe QTL on chromosome 3 were used to query the soybean genome to identify the syntenic region on chromosome 19. Genes within QTL were plotted using ggplot2 (Wickham 2016).

## Results

### Phenotypic results

To allow phenotypic changes resulting from brief periods of nutrient stress to manifest, phenotyping data was collected 7 days after T2, when the 6th trifoliate had developed and emerged. Plants exposed to either –Fe or –P_i_T1T2 stress exhibited larger 6th trifoliates than plants grown in controlled conditions and plants exposed to stress only at T2 (Fig. 2a). Plants exposed to either –Fe or –P_i_ stress at T2 showed statistically significant increases in both root and shoot weights (Fig. 2b, c). Shoot diameter was unaffected by stress nutrient stress exposures (Fig. 2d). Both –Fe and –P_i_ stresses applied at T1T2 and T2 resulted in improved SPAD readings of the 6th trifoliate on day 7 compared to plants grown consistently in sufficient conditions (Fig. 2e). These combined results indicate that stress exposure improves plant fitness in Clark, though there is no indication from the phenotypic measurements that repeated stress exposure increases plant fitness over a single stress event.

Phenotypic changes are a result of gene expression changes in response to treatment. Using RNA-seq data, we were able to identify genes differentially expressed due to either Fe or P_i_ deficiencies (–FeT1, –FeT1Rec, –FeT2, –FeT1T2, –P_i_T1, –P_i_T1Rec, –P_i_T2, –P_i_T1T2) by comparing to nutrient sufficient controls harvested at the same timepoint (ControlT1, ControlT1Rec, and ControlT2, Online Resources 5 and 6). In total, we identified 7866 and 13,770 genes responding to –Fe stress in leaves and roots, respectively (Table 1). Similarly, we identified 7198 and 17,298 differentially expressed genes in –P_i_-stressed leaves and roots, respectively (Table 1).

**Table 1:** Distribution of differentially expressed genes

Tissue	Time	Fe		P_i_	
		Down	Up	Down	Up
Leaves	T1	3314	1990	20	279
	T1Rec	897	1492	1589	2564
	T1T2	96	120	277	627
	T2	881	950	1091	2393
Roots	T1	3746	1965	179	1018
	T1Rec	29	472	6058	6537
	T1T2	3905	6388	3816	6270
	T2	3850	6249	3788	6032

A total of 7866 and 7198 DEGs were identified in the leaves of –Fe- and –P_i_-stressed plants, respectively. Similarly, a total of 13,770 and 17,298 DEGs were identified in the roots of –Fe- and –P_i_-stressed plants, respectively. Genes may be differentially expressed in both tissues, at multiple timepoints, and may be shared by both the –Fe and –P_i_ stress responses

### Confirming gene expression patterns

The qRT-PCR analysis tested 16 genes in 70 unique gene, nutrient stress, and timepoint combinations (Online Resources 7 and 8). This analysis confirmed the direction of gene expression for 90% of the combinations tested with a correlation, as calculated by Microsoft Excel, between the qRT-PCR and RNA-seq of 80%. The *R*^2^ value of a linear regression line for all 70 reactions was 0.6428. This is explained by the expression profiles of the RNA-seq data being greater than those measured by qRT-PCR at 74% of the time. This is likely due to the RNA-seq data utilizing uniquely mapping reads. Overall, the qPCR results confirm the expression profiles measured by RNA-seq analysis.

### Gene expression patterns in response to nutrient stress

Comparing the number of DEGs within and between nutrient-stressed plants at each timepoint (Table 1) revealed important biological insights. After an initial stress (T1), soybean leaves responded to a lack of available iron by altering the expression of 5304 genes, the majority of which were downregulated. In comparison, soybean leaves failed to respond to a lack of available phosphorus at T1, only altering the expression of 299 genes, with the majority (93%) upregulated. The same response is observed in roots with 65% of the 5711 differentially expressed genes downregulated in response to –Fe stress and 85% of the 1197 differentially expressed genes upregulated in response to –P_i_ stress. When plants were given 48 h to recover from nutrient stress (T1Rec), 2389 and 501 genes were differentially expressed in –Fe recovery leaves and roots, respectively, when compared to plants grown in nutrient-sufficient conditions. In contrast, following –P_i_ recovery, 4153 and 12,595 genes were differentially expressed in leaves and roots, respectively. At T2, leaves from plants exposed to single or multiple exposures of –Fe stress exhibit similar expression patterns, but more genes were differentially expressed in –FeT2 (1831) than in –FeT1T2 (216). The same pattern is observed in leaves of –P_i_-stressed plants; 3484 DEGs identified in –P_i_T2 leaves while only 904 DEGs were identified in –P_i_T1T2 leaves. In roots of the same plants, 10,099 DEGs were identified in –FeT2 and 10,243 DEGs in –FeT1T2, similarly 9820 DEGs in –P_i_T2 and 10,086 DEGs in –P_i_T1T2.

To visualize changes in gene expression across time, we combined all DEGs responding to –Fe or –P_i_ stress in each tissue. Using hierarchical clustering, we identified clusters of genes with similar expression patterns across treatments in each tissue. In –Fe-stressed leaves, the 7866 DEGs clustered into seven distinct groups (Online Resource 5, Fig. 3a, FeL1–FeL7). The largest number of DEGs was observed at T1 and in T1Rec. Expression patterns of T2 and T1T2 samples are very similar, but expression is opposite of that measured at T1. In –Fe roots, the 13,770 DEGs clustered into six expression clusters (Online Resource 6, Fig. 3b, FeR1–FeR6). Roots had a strong response to the initial iron stress, but after recovery, few genes remained differentially expressed. Expression in T2 and T1T2 was remarkably similar, but the direction of gene expression was reversed compared to that in T1, just as was observed in the leaf data.

**Fig. 3:**
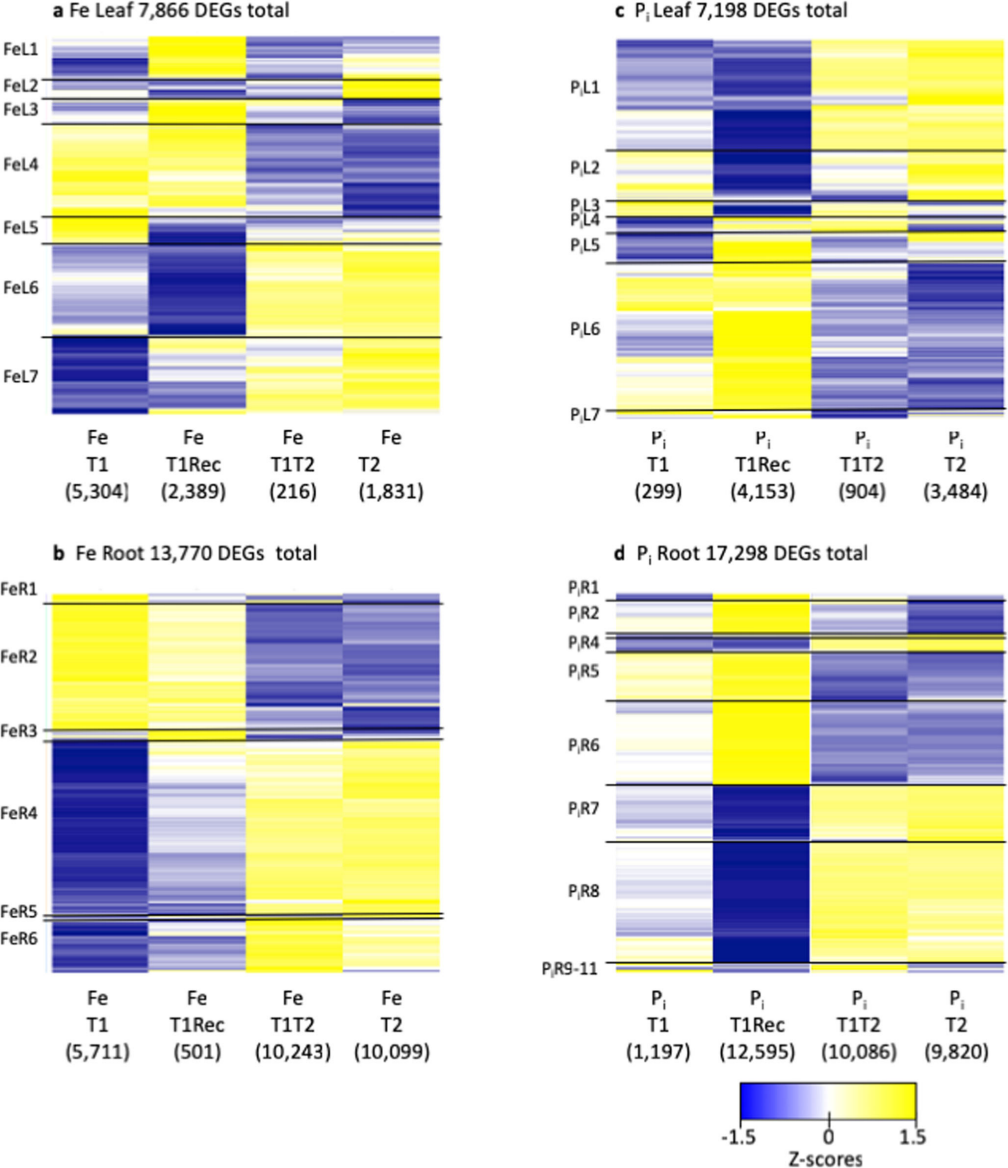
Gene expression of all differentially expressed genes identified. Expression profiles (as *Z* scores) of all differentially expressed genes from –Fe leaves (**a**) and roots (**b**) and from –P_i_ leaves (**c**) and roots (**d**). Genes upregulated compared to control plants are yellow, and those downregulated compared to control are blue. The total number of genes differentially expressed in each tissue is provided above each heatmap while the number of differentially expressed genes at each timepoint is provided in parentheses below each column. Genes sharing similar expression patterns were identified, and gene cluster designations are provided to the left of each heatmap with the boundaries of each cluster denoted by horizontal black lines

Examining the DEGs from –P_i_-stressed plants revealed a completely different gene expression pattern; few DEGs responded to the initial –P_i_ stress (–P_i_T1), instead the majority of genes responded to phosphate resupply (–P_i_T1Rec). In leaves, the 7198 DEGs identified from –P_i_ leaves clustered into seven groups (Online Resource 5, Fig. 3c, P_i_L1–P_i_L7). As was observed in –Fe leaves, the gene expression at T1T2 and T2 (when plants are 18 days post germination) was opposite of that measured at T1 (when plants are 14 days post germination). In –P_i_ roots, the pattern was even more dramatic with only 1197 DEGs at T1 but 12,595 DEGs at T1Rec (Fig. 3d). The 17,298 genes differentially expressed in roots due to –P_i_ stress clustered into 11 groups (Online Resource 6, PiR1–P1R11). As was observed in –Fe plants and –P_i_ leaves, the expression patterns of T1T2 and T2 are nearly identical, but the direction of expression is opposite of that observed at T1Rec. Given that all the plants in this study were grown simultaneously, our data suggests that collectively across treatments and tissues, the age of the plant (14 days to 18 days) is one of the biggest factors in determining gene expression changes in response to single or multiple stress exposures.

### Characterizing genetic networks responding to nutrient stress

**Fig. 4:**
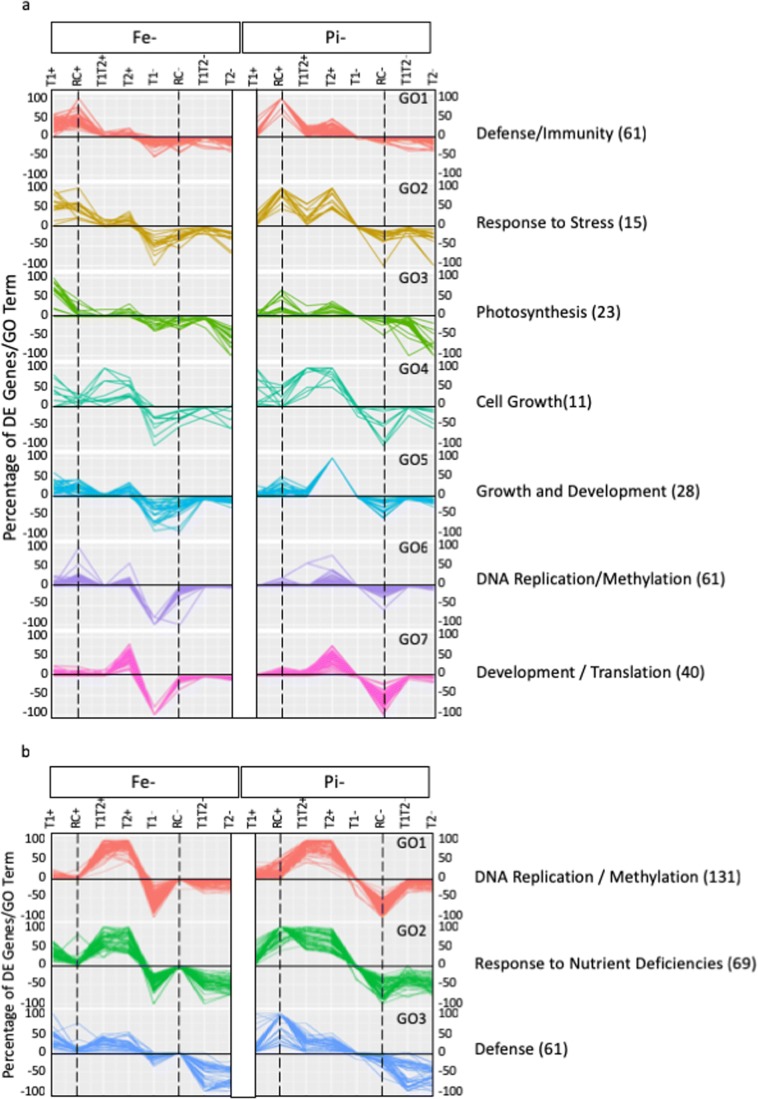
Prevalence and pattern of overrepresented Gene Ontology (GO) terms. The percentage of differentially expressed genes at each timepoint associated with GO clusters. Seven clusters were identified for overrepresented GO terms from leaves (**a**), and three were identified for terms from roots (**b**). Each line represents a unique GO term overrepresented in at least one of the timepoints. Biological processes were assigned to each cluster based on the prevalence of the top five (leaves) or ten (roots) overrepresented GO terms for each cluster. These terms are provided on the right. The total number of overrepresented GO terms within each cluster is provided in parentheses following the cluster annotation. Unique patterns for –Fe and –P_i_ deficiency stress and the shift in timing of DEGs associated with each GO term can be observed. Vertical dashed lines highlight the T1Rec timepoints to assist with between-clusters comparisons

To allow comparisons of the pathways responding to –Fe and –P_i_ stress, we identified significantly overrepresented (corrected *P* < 0.05) GO terms from each of the DEG treatments and timepoints (Fig. 4, Online Resource 9). This approach identified 403 unique GO terms, 239 from leaves and 261 from roots with 152 GO terms common between one or more timepoints in roots and leaves. For each significant and unique GO term within a tissue, we determined the number of DEGs in that GO term differentially expressed at each timepoint. We then divided this number by the total number of genes in the genome associated with that GO term. Using percentages instead of actual DEGs allowed small GO terms to have equal representation with large GO terms. We then clustered this information across all GO terms to identify GO terms with similar expression patterns across treatments and timepoints. Using this approach, we identified seven unique GO patterns in leaves (Fig. 4a) and three in roots (Fig. 4b). In the leaves, GO clusters contained between 11 and 61 GO terms: defense/immunity (61), response to stress (15), photosynthesis (23), cell growth (11), growth and development (28), DNA replication/methylation (61), and development/translation (40). Similarly, in roots, GO clusters contained between 61 and 131 GO terms: DNA replication/methylation (131), response to nutrient deficiencies (69), and defense (61). These results suggest that roots quickly adapt to the changing nutrient status and achieve a new homeostatic level within 24 h. In contrast, the leaves exhibit more variability, suggesting that nutrient status signaling takes longer to reach the leaves and that the leaves may induce multiple responses to counter aberrations in nutrient availability. Biological functions were assigned to each of the GO clusters by examining the annotations of the five most prevalent GO terms in leaves and ten most prevalent GO terms in roots. Biological processes associated with these GO annotations were obvious. In leaves, genes in clusters 1 and 2 were associated with GO terms involved in defense, with most DEGs from –P_i_T1Rec. GO terms for cluster 3 were associated with photosynthesis, and GO terms for clusters 4 and 5 were associated with growth. Finally, clusters 6 and 7 were primarily associated with DEGs from –FeT1 and were associated with GO terms for DNA replication and methylation. In roots, cluster 1 was associated with DNA replication and methylation, cluster 2 was involved in stress responses and growth, and DEGs in cluster 3 were associated with defense responses.

**Fig. 5:**
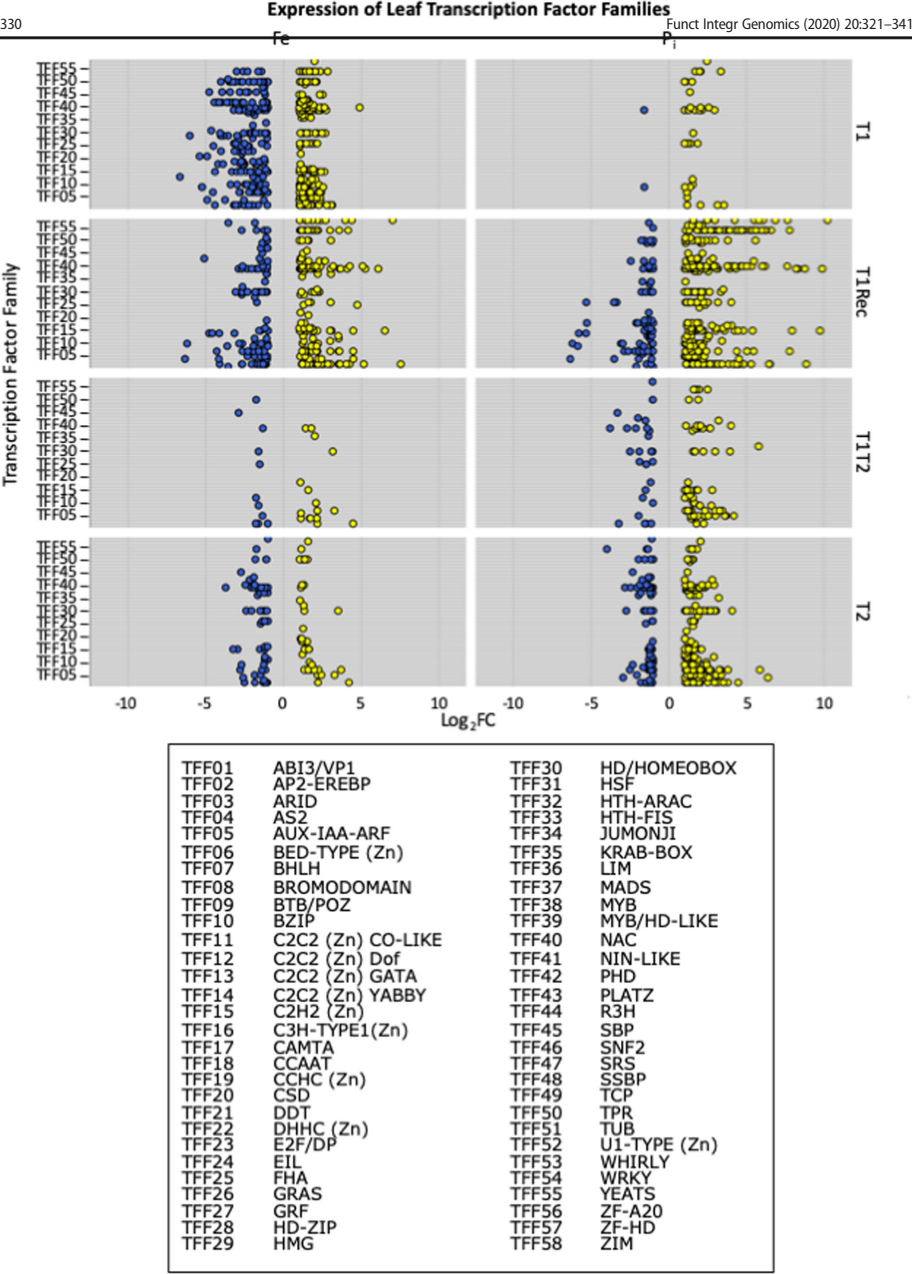
Expression of leaf transcription factors (TFs) by transcription factor family (TFF). All differentially expressed TFs were identified at each timepoint and classified based on TFF. Upregulated TFs are in yellow, and downregulated TFs are in blue. Expression is presented as Log2 fold change (Log2FC) on the *X*-axis

**Fig. 6:**
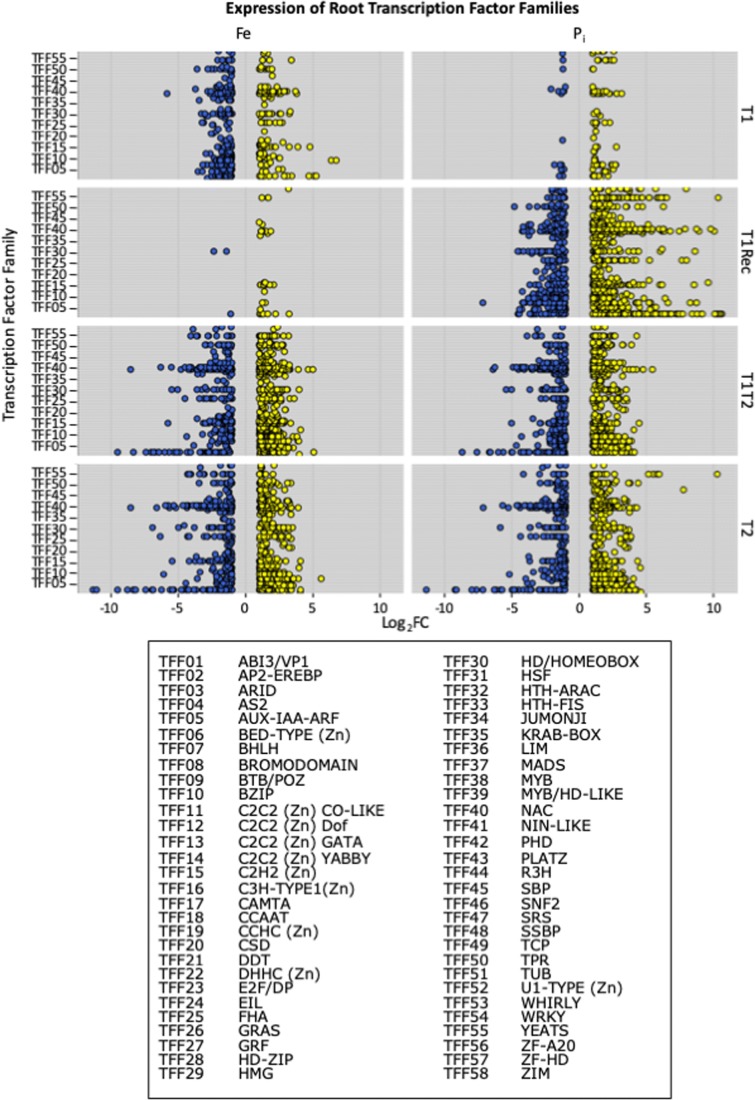
Expression of root transcription factors (TFs) by transcription factor family (TFF). All differentially expressed TFs were identified at each timepoint and classified based on TFF. Upregulated TFs are in yellow, and downregulated TFs are in blue. Expression is presented as Log2 fold change (Log2FC) on the *X*-axis

To identify TFs regulating gene expression important for both –Fe and –P_i_ stress responses in soybean, we identified differentially expressed TFs at each timepoint and graphed their expression by TFF in leaves and roots by collection time (Figs. 5 and 6). For both leaves and roots, the expression pattern of TFs directly mirrored the expression pattern of all DEGs. However, statistical analyses identified eight TFFs significantly overrepresented in leaves (AP2-EREB, AUX-IAA-ARF, E2F-DP, homeodomain, NAC, TPR, WRKY, ZIM) and seven significantly overrepresented TFFs in roots (AP2-EREB, AUX-IAA-ARF, GRAS, homeodomain, NAC, WRKY, ZIM) at individual timepoints, representing 254 differentially expressed (DE) TFs (Table 2, Online Resource 10). In roots, the NAC TFF was overrepresented at all timepoints except –FeT1Rec and –P_i_T1. Other TFFs were only significant at specific timepoints. In leaves, far fewer DE TFs were identified, mirroring the overall DEG expression pattern. However, fewer DE TFs made it easier to highlight expression pattern differences. Also evident in the leaves is the increased number of TFFs and more TFs within each TFF represented at the T2 timepoint compared to the T1T2 timepoint. This suggests the initial stress event represses TF expression at T1T2. Interestingly, this is only evident in the leaves, not in the roots. This could be because roots are responsible for nutrient sensing and uptake while gene expression levels in leaves might simply change in response to available nutrients.

**Table 2:** Overrepresented transcription factor families

TFF	Leaf								Root							
	FeT1	FeT1Rec	FeT1T2	FeT2	P_i_T1	P_i_T1Rec	P_i_T1T2	P_i_T2	FeT1	FeT1Rec	FeT1T2	FeT2	P_i_T1	P_i_T1Rec	P_i_T1T2	P_i_T2
AP2-EREBP (343)		44										85		136	99	99
AUX-IAA-ARF (117)							11					34		42	36	
E2F/DP (12)	7															
GRAS (113)											35			47		
Homeodomain/HOMEOBOX (274)								37			71	68				
NAC (187)						37			37		62	61		71	66	59
TPR (279)	47															
WRKY (174)		35				51								62		
ZIM (24)		10				10							9	17		

The number of transcription factors (TFs) within each transcription factor family (TFF) differentially expressed at each timepoint in roots and leaves was compared to the total number of TFs in that TFF in the genome (provided in parentheses after the TFF name). A Bonferroni correction was applied to correct for overtesting (corrected *P* value < 0.05). These analyses identified a total of 254 unique DE TFs belonging to the overrepresented TFFs

### Identifying nutrient stress memory genes in soybean

Once plants experience stressful growth conditions, it might be expected that most genes would respond similarly to a second stress exposure. However, some genes may respond differently to a second stress compared to an initial stress exposure. To identify these *memory* genes, we must be able to compare plants of the same age that have experienced different numbers of stress events. In our analyses, we compared the T1T2 and T2 plants in order to identify two kinds of memory genes: those expressed in opposite directions between a first and second stress exposure and those differentially expressed only after the second stress exposure. In the leaves of –Fe-stressed plants, 63 genes with opposite expression patterns between T1T2 and T2 were identified. An additional 274 were identified from –P_i_-stressed leaves. Clustering analysis resulted in a heatmap of 333 unique DEGs organized into four clusters (Online Resources 11 and 12a). Performing the same analyses for root DEGs identified 293 genes from –Fe-stressed plants and 235 genes from –P_i_-stressed plants. Visualizing expression profiles of these 486 genes results in a heatmap with five unique clusters (Online Resources 11 and 12b). Over 20% of these genes were homologous to *memory genes* in *Arabidopsis* (Ding et al. 2013; Liu et al. 2014). We identified a total of 3128 DEGs in roots and 247 DEGs in leaves that were unique to the T1T2 timepoint (Online Resource 13). These genes represent genes differentially expressed after a second exposure that were not differentially expressed in response to an initial stress exposure. Of the 3128 DEGs in roots, 1247 were identified in –P_i_-stressed plants while 2212 were identified in –Fe-stressed roots, with 331 common to both nutrient deficiencies. In leaves, only 59 memory DEGs were identified in –Fe-stressed plants and 193 in –P_i_-stressed plants, with five genes common to both nutrient deficiencies. Comparing these genes to memory genes identified by Ding et al. (2013) found 32% of the genes in roots and 36% of the genes in leaves in common. This conservation between species and different stresses suggests these genes may be part of a core set of stress response genes. The remaining genes indicate there may be unique gene expression responses for each species and/or each stress condition.

### Identifying candidate genes within nutrient stress QTL

Despite the critical need to understand how soybean and other crops adapt to –P_i_ stress, only a few studies have investigated P_i_ deficiency in soybean. Further, a major criticism of gene expression studies is that they do not correlate well with genetic studies. Therefore, we mined the available literature for known –P_i_ QTL in soybean. Multiple studies from China have identified P_i_-associated QTLs (Liang et al. 2010; Ning et al. 2016; Zhang et al. 2009, 2014, 2017). These studies have identified a cluster of QTLs on MLG D1b+W (now chromosome 2) responsible for 74% of the phenotypic variations of –P_i_ stress traits and a QTL on chromosome 8 which explains up to 41% of the phenotypic variations (Zhang et al. 2009, 2014). Minor P_i_ QTLs were identified on chromosomes 4, 14, and 18. Additionally, SNPs associated with –P_i_ tolerance have been identified and two RNA-seq studies of –P_i_-stressed soybeans were recently published: one using Williams 82 and the other using P_i_-efficient and P_i_-inefficient lines from China (Zhang et al. 2016a, 2017). We associated marker information from these studies with gene intervals within the soybean genome, allowing us to overlay our differentially expressed genes across the genomic regions identified by QTL and GWAS analyses. We could then use this approach to identify candidate genes underlying the QTL regions.

The –P_i_ tolerance QTL on chromosome 2 corresponds to a genomic region encoding 115 genes (*Glyma.02G268000*–*Glyma.02G256700*), 52 and 50 of which were differentially expressed in response to –P_i_ stress and –Fe stress, respectively (Online Resource 14a). Among these genes are two phospholipase genes (*Glyma.02G257000*–*Glyma.02G257200*) and a ferritin gene (*Glyma.02G262500*). The work by Bournier et al. (2013) demonstrated that under –P_i_ growth conditions, *AtFER1* expression is induced by AtPHR1, a phosphate starvation response TF. In *Arabidopsis*, these phospholipase genes are involved in signal transduction by regulating phosphoinositide, a rapidly defusing signal molecule that responds to environmental conditions and regulates auxin and abscisic acid (ABA) biosynthesis (Wang et al. 2006; Shewan et al. 2011). In the middle of the QTL, one of the most differentially expressed genes in the QTL is *Glyma.02G261400*, which encodes a leucine-rich receptor-like kinase whose homolog in *Arabidopsis* is upregulated by multiple abiotic stresses and is involved in hormone and abiotic stress signal transduction (ten Hove et al. 2011). Additionally, the two genes at the end of the QTL encode a dual specificity phosphatase and a gene homologous to ACO4. These genes are upregulated in leaf tissues and highly upregulated at –FeT1 and –P_i_T1Rec in roots but significantly downregulated at both –Fe and –P_i_ at T1T2 and T2. ACO4 is an important enzyme in ethylene formation. This supports previous findings of increased ethylene production in roots of –P_i_-stressed plants which is known to alter the root architecture and the activity levels of P_i_ transporters and acid phosphatases in response to –P_i_ stress (Nagarajan and Smith 2012; Song and Liu 2015).

The QTL on chromosome 8 corresponds to a genomic region encoding 27 predicted genes (Online Resource 14b), 15 of which are differentially expressed by either –Fe or –P_i_ stress. Among the differentially expressed genes, three encode acid phosphatases (*Glyma.08G194900*–*Glyma.08G195100*), including the acid phosphatase recently identified using RNA-seq by Zhang et al. (2017). Overall, the acid phosphatase genes are highly upregulated in –P_i_-stressed leaves and roots, while their expression in –Fe-stressed tissues is varied.

Finally, the QTL region identified on chromosome 18 (Online Resource 14c) spans 79 genes and contains *Glyma.18G200500*, which is downregulated in –P_i_ T1Rec leaves. The *Arabidopsis* homolog of this gene (*AT3G51860*) mediates a shoot-derived signal that modulates the activity of root PHT1 P_i_ transport system and *SPX1* and *SPX3* genes (Liu et al. 2011). SPX proteins regulate P_i_ starvation responses and signaling in both monocots (rice) and dicots (white lupin) (Wang et al. 2014; Zhou et al. 2015). Their role in the soybean P_i_ signaling was recently confirmed (Zhang et al. 2016a, b). However, the most differentially expressed gene in the QTL is *Glyma.18G204000* which encodes a heat shock protein. This protein is known to play a role in thermotolerance, likely by refolding or degrading unfolded or misfolded proteins (Ma et al. 2015). The altered expression in roots of both –Fe- and –P_i_-stressed plants suggests it plays a similar role in nutrient deficiency stress tolerance. Also highly differentially expressed in roots is *Glyma.18G205400*, with no known function, and *Glyma.18G206600*, the homolog of ERF48. In *Arabidopsis*, this gene is involved in the abscisic acid signaling to enhance tolerance to oxidative stresses including heat and salt stress (Yang et al. 2013; Song et al. 2014). Finally, four sequential genes (*Glyma.18G204300*–*Glyma.18G204600*), all encoding receptor-like proteins, are upregulated in –P_i_ T1Rec samples. Receptor-like proteins are known to regulate development and defense responses (Gust and Felix 2014), possibly altering developmental processes in response to nutrient deficiencies. In rice, the RLP gene *OsRMC* is involved in regulating iron acquisition and is upregulated in response to –P_i_ (Yang et al. 2013), suggesting conserved responses to the micro- and macronutrients.

## Discussion

### Nutrient deficiencies in soybean

Iron and phosphate are both recognized as essential nutrients for plant growth. In the Upper Midwest, iron deficiency is a perennial problem resulting in 120 million USD in yield loss each year (Hansen et al. 2004). Due to soybeans’ economic importance, there has been a suite of studies identifying genomic regions of interest (Lin et al. 1997, 2000; Severin et al. 2010; Peiffer et al. 2012; Mamidi et al. 2014) and molecular networks regulating iron uptake, utilization, homeostasis, and low iron tolerance (O’Rourke et al. 2009; Atwood et al. 2014; Moran Lauter et al. 2014). These studies have identified key steps in the iron-responsive molecular networks for crop species. Specifically, genes and pathways involved in DNA replication/methylation, iron uptake, and defense are critical components of the soybean iron deficiency response. In contrast, the role of phosphate has not been similarly investigated in soybean. Phosphate fertilizer is commonly applied to fields across the Upper Midwest, eliminating phosphate deficiency as a problem for growers. However, improved phosphate acquisition and utilization efficiency is critically important for yield preservation, fiscal feasibility, and environmental sustainability of US farming systems, especially as rock phosphate reserves are expected to be depleted in the next 50 years (Cordell et al. 2009). Understanding the molecular pathways and networks underlying micro- and macronutrient uptake, utilization, and homeostasis in soybean provides a foundation for plant improvement through traditional breeding and cutting-edge molecular approaches.

Multiple studies have shown evidence of macronutrient (P_i_) and micronutrient (Fe) crosstalk (Zheng et al. 2009; Li and Lan 2015; Rai et al. 2015). Additionally, previous studies have shown that altering P_i_ availability impacts iron homeostasis; low –P_i_ conditions can mimic Fe toxicity. This includes altering the transcriptional responses of genes involved in iron homeostasis, storage, and transport (Hirsch et al. 2006; Bournier et al. 2013). These studies illustrate a molecular link between iron and phosphate deficiency worthy of further investigation.

For the first time, we have directly compared whole-genome expression responses to micro- and macronutrient deficiencies in soybean, allowing us to take note of several important trends.

### Speed and diversity of the soybean nutrient stress response

One of the first trends we observed was that soybean responds quickly to changes in nutrient availability. After 24 h, –Fe and P_i_ stress resulted in the differential expression of over 10,000 genes in roots and leaves at T1 and T2. In contrast, after 24 h of –Fe stress in *Arabidopsis* roots, Stein and Waters (2012) identified 821 and 394 DEGs in *Arabidopsis* ecotypes Kas-1 and Tsu-1, respectively. Analysis of rosettes of the same plants identified 71 and 616 DEGs (Waters et al. 2012) While previous work from our group has demonstrated that soybean responds to iron stress in as little as 1 h (Moran Lauter et al. 2014), this study was the first to demonstrate the speed and diversity of the soybean stress response to multiple nutrient deficiencies and multiple stress events.

### Soybean responds to Fe deficiency and P_i_ availability

Our second observation was that soybean responds to Fe deficiency, but P_i_ sufficiency. At –FeT1, we observed 5304 and 5711 DEGs in leaves and roots, respectively. At –FeT1Rec, 48 h after iron stress recovery, only 2389 and 508 genes were differentially expressed in the same tissues. If we compare –FeT1 and –FeT1Rec in the heatmaps in Fig. 3a and b, almost all genes in the roots are expressed at lower levels following recovery, as are many of the genes in the leaves. For P_i_ stress, the opposite pattern emerges. At –P_i_T1, 279 and 1197 genes were differentially expressed in leaves and roots, respectively. At –P_i_T1Rec, this number dramatically increases to 4153 and 12,595 DEGs in leaves and roots, respectively. Examining the heatmaps in Fig. 3c and d, it is clear the strongest differential expression is associated with phosphate resupply.

### Plant age affects the soybean nutrient stress response

Our third observation was that the age of the plant could impact nutrient stress responses. In our experiment, plants at T2 were 3 days older than plants at T1 when encountering their first stress. For –Fe-stressed leaves, greater numbers of DEGs were identified at –FeT1 compared to –FeT2 (Table 1). There were no genes in common between –FeT1 and –FeT2. For –Fe-stressed roots, more DEGs were identified at –FeT2, with only 20 DEGs in common between –FeT1 and –FeT2 roots. However, when we ignored the direction of expression, we identified 1122 and 4379 common genes between T1 and T2 in leaves and roots, respectively, suggesting many genes had flipped their direction of expression between T1 and T2. While a large number of differentially expressed genes were uniquely differentially expressed in T1 or T2, we can observe in Fig. 3a and b that the majority of genes change expression patterns between T1 and T2, suggesting a conserved gene repertoire but slight differences in the timing of the iron stress response as the plants aged.

The same patterns did not hold true for –P_i_ stress. In leaves and roots, 89 and 536 DEGs, respectively, were common to –P_i_T1 and –P_i_T2. Of these genes conserved between –PiT1 and –PiT2, only 8 and 87 DEGs in leaves and roots, respectively, flipped direction of expression between –P_i_T1 and –P_i_T2. Unlike the –Fe response, most DEGs in common between timepoints maintained their direction of expression. In addition, many more DEGs were identified at –P_i_T2 in both leaves and roots (3484 and 9820 DEGs, respectively), suggesting a stronger, more dynamic response to –P_i_ stress as the plants aged.

### Fe and P_i_ stress responses use the same signaling networks

Fourth, we observed that –Fe and –P_i_ stress signaling uses the same molecular pathways. Over all genes differentially expressed in response to –Fe or –P_i_ in the root, 11,711 are in common (85% of –Fe-responsive genes and 68% all P_i_-responsive genes). In contrast, only 3512 genes were in common between –Fe (45%) and –P_i_ (51%) in leaves. For each timepoint and sample, we identified significantly overrepresented Gene Ontology biological process terms. For each unique significant GO term, we then tracked the number of DEGs per timepoint and sample. We then clustered this data to identify GO terms, with similar expression patterns across our datasets. In roots, we identified three GO clusters associated with gene silencing/DNA replication and stress responses including responses to iron and phosphate starvation and defense (Fig. 4b). Other than differences in timing due to phosphate resupply, –Fe and –P_i_ responses look remarkably similar. In leaves, we identified seven GO clusters (Fig. 4a). These clusters were associated with defense/immunity, response to stress, photosynthesis, development, cell wall modifications, and gene silencing/DNA replication and translation. Like the roots, we observed differences in responses to iron stress and phosphate resupply. However, we observed other differences in the leaves that may impact how each of these nutrients is stored and mobilized in the leaves. Interestingly, for leaf GO clusters 1 and 6, more than half of the GO terms in the cluster had more DEGs at FeT1Rec+ than at –FeT1+, mirroring the –P_i_ recovery response. Leaf GO cluster 6 was also notable for a strong peak at –FeT1−, not observed in –P_i_T1− or –P_i_T1Rec−. Similarly, leaf GO clusters 2 and 5 have strong peaks at –P_i_T2+, not observed in –FeT2+.

These findings suggest striking similarities in mechanisms used for Fe and P_i_ uptake by the roots, but differences in how available nutrients are stored and utilized in the leaves. Excess micro- and macronutrients are stored within the plant to avoid toxicity and the formation of damaging free radicles. Excess macronutrients are usually stored in the vacuole while over 80% of the Fe (micronutrient) is found in chloroplasts and ferritin proteins. Additional micro- and macronutrients may be bound within the root apoplasmic spaces, which bind micronutrient cations tighter than macronutrient cations. Differential expression of genes associated with the response to phosphate starvation, cell wall modification, and galactolipid biosynthesis was a component of soybean leaf GO clusters 4 and 5 (Fig. 4a). Previous work has shown that biosynthesis of galactolipids, which are non-phosphorus lipids, is induced by –P_i_ deficiency as a mechanism to conserve and recycle existing P_i_ (Härtel et al. 2000; Geske et al. 2013). These GO categories support the hypothesis that the early –P_i_ stress response is to utilize stored P_i_ from vacuoles and reorganize cell walls and membranes to release integrated P_i_. The P_i_ stored within the plant is sufficient to maintain a relatively homeostatic state with little to no effect on DNA replication and plant growth and development, at least for the short-term stress of our study. Upon P_i_ resupply, a massive transcriptional shift occurs as the plant increases nutrient uptake to restore normal P_i_ stores. P_i_ resupply also induces a large number of genes involved in defense responses that were not affected by –P_i_ conditions. It is possible under –P_i_ stress the plant is more susceptible to pathogen infection and additional abiotic stresses, and upon returning to normal P_i_ conditions, the plant mounts a delayed defense response to address damage incurred during –P_i_ stress. In contrast to the large amount of P_i_ stored within the plant, there is relatively little stored Fe. Thus, when moved to low-Fe conditions, the plant immediately induces a massive transcriptional shift that induces both iron-specific and broad stress responses.

### Distinct transcription factors regulate timing and diversity of stress responses

Within each timepoint and sample, we identified significantly overrepresented TFFs regulating complex molecular signaling cascades and dynamic changes in gene expression. Consistent with previous statements, there were more TFFs overrepresented in leaves (eight) than in roots (seven). All of the overrepresented TFFs have been associated with multiple biological processes and responses. In leaves, members of the TPR TFF modulate auxin and jasmonic acid signaling, intracellular pH, thermotolerance, the *Pseudomonas syringae* defense response, and link the response to DNA damage to epigenetic silencing (Bissoli et al. 2012; Causier et al. 2012; Pogorelko et al. 2014). The E2F TFF is best known for regulating cell cycle (Wang et al. 2018). O’Rourke et al. (2009) and Atwood et al. (2014) both proposed the involvement of E2F transcription factors in regulating the soybean iron stress response. Atypical members of this TFF, which include the three DE soybean genes *Glyma.06G086800*, *Glyma.05G033400*, and *Glyma.17G093600*, are also important in regulating endoreduplication, which has been associated with increased stress tolerance (Lammens et al. 2009; Heckmann et al. 2011; Radziejwoski et al. 2011). The ZIM TFF has previously been associated with hormone (JA) signaling and may regulate the trade-off between growth and defense responses to abiotic stress through interactions with MYC TFs (Chung and Howe 2009; Major et al. 2017). We identified a number of MYC TFs that were DE, but not significantly overrepresented at any timepoint in either roots or leaves. In roots, NAC TFs play a pivotal role in abiotic stress response networks conferring tolerance to multiple abiotic stresses, regulating abscisic acid and salicylic acid biosynthesis, and mediating stress response and proteasome stress networks (Shao et al. 2015; Welner et al. 2015; Zheng et al. 2015; Gladman et al. 2016; Liu et al. 2017). Finally, the GRAS TFF is important for root growth responses to environmental stress and has been found to play an important role in maintaining root plasticity (Fode et al. 2008; Yuan et al. b; Choe et al. 2017).

**Fig. 7:**
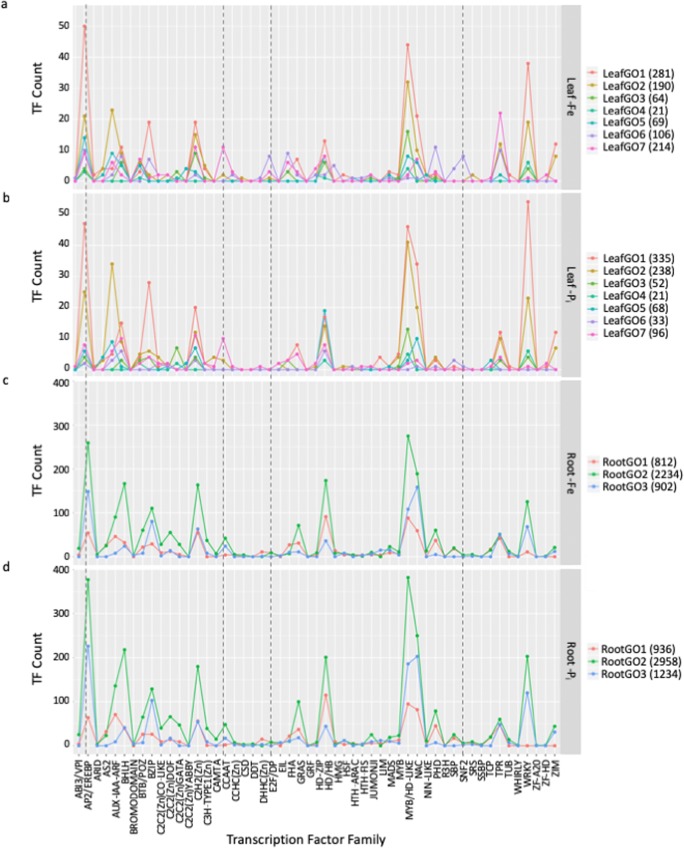
Identifying transcription factors (TFs) associated with Gene Ontology (GO) terms. The number of differentially expressed TFs by transcription factor family (TFF) and GO cluster in leaves and roots The color of the lines corresponds to the GO clusters in Fig. 4. This analysis identifies individual TFs from a single TFF associated with different biological processes as described by the GO clusters. TFFs present in –Fe leaves, but not –P_i_ leaves include SNF2 and E2F (highlighted with a vertical dashed line). More TFs are DE in roots, but patterns between –Fe roots and –P_i_ roots are similar

Our analysis identified a total of 1995 unique TFs DE in roots and 1127 in leaves. The overrepresented TFF analyses only represented 254 DE TFs, ignoring the majority of DE TFs. To investigate additional DE TFs, we extracted differentially expressed TFs from the overrepresented GO clusters described above, representing 652 of the DE TFs in leaves and 1647 of the DE TFs in roots, graphing the number of DE TFs by TFF and associated GO cluster (Fig. 7). This revealed important discrepancies between leaves and roots and between –Fe- and –P_i_-stressed plants. In leaves, expression profiles between –Fe- and –P_i_-stressed plants (Fig. 7a) were different. The E2F and SNF2 TFFs appear unique to the –Fe-stressed leaves with few or no members of these TFFs differentially expressed at any timepoint in –P_i_-stressed leaves. Assigning the individual members of TFFs to GO clusters allowed us, for the first time, to visualize the breakdown of TFFs by biological function. The prevalence of TFs within a TFF in each GO cluster is different. For example, in leaves, AP2/EREBP TFs are most associated with GO1, which is involved in defense and immunity, while CCAAT TFs are most associated with GO7, which is involved in development and translation. While a gene may have multiple GO annotations, few of the TFs are represented in more than 1 GO cluster, meaning each peak represents a unique repertoire of TFs associated with each biological process.

Finally, we used the *Arabidopsis* homologs of the differentially expressed TFs to graph known interactions using the STRING database (Szklarczyk et al. 2017). Combining this information with the previously described GO analysis allowed us to identify unique clusters of TFs, often from multiple TFFs, which interact to regulate specific biological processes (Online Resource 15). TFs differentially expressed in leaves (Online Resource 15a) were highly interconnected with TFs from multiple GO clusters represented while the TFs in roots (Online Resource 15b) were less connected. Groups of highly networked TFs in roots were often anchored by a single *hub* gene, likely involved in regulating the expression of other genes in the cluster. These analyses provide novel insight into how specific TFs from various TFFs interact to regulate interconnected and diverse responses to nutrient deficiencies. This information will be leveraged in future experiments to investigate the role of specific biological pathways under nutrient deficiency stress.

### Does an initial stress event alter gene expression responses to a second stress event?

Previous studies in *Arabidopsis* identified 1291 genes expressed in the opposite direction after three rounds of dehydration stress compared to expression after a single stress exposure (Ding et al. 2013). In our study, we identified two kinds of memory genes: those where the direction of expression changed between T1 and T1T2 and those only differentially expressed after a second stress exposure. We identified 819 genes from leaves and roots of soybean plants exposed to two stress events that were expressed in the opposite direction compared to after a single stress event. These 819 soybean genes correspond to 567 unique *Arabidopsis* genes. Comparing the *Arabidopsis* genes to the memory genes identified by Ding et al. (2013) found 74 genes common to both experiments (Online Resource 11). These genes were involved in a variety of biological processes including defense, as evidenced by the identification of leucine-rich repeat protein (*AT1G03440*, *AT2G31880*) and receptor-like kinase (*AT1G70520*, *AT4G23180*) genes. Also represented in both studies were genes associated with hormone biosynthesis pathways. DAR2 (AT2G39830) coordinates the cytokinin and auxin crosstalk, JAR1 (AT2G46370) is involved in jasmonic acid (JA) biosynthesis, and two genes (*AT4G24380* and *AT5G38710*) were associated with ethylene biosynthesis in –P_i_-stressed lupin roots (O’Rourke et al. 2013). Additionally, three AtCMPG1 homologs, which possess the four conserved amino acid residues: Cys, Met, Pro, and Gly (*Glyma.11G214500*, *Glyma.02G242900*, and *Glyma.14G212200*), were associated with increased drought tolerance but were also identified as memory genes in response to –Fe stress. In *Arabidopsis*, JA biosynthesis and signaling were induced by –P_i_ deficiency in both roots and leaves, leading to reduced growth in aboveground tissues, anthocyanin accumulation in leaves, and defense responses (Khan et al. 2016). Two genes *Glyma.09G071600* and *Glyma.01G204400*, homologs of the *Arabidopsis* genes *JAZ1* and *TIFY10*, respectively, were identified as memory genes in –P_i_ roots. JAZ1 and TIFY10 regulate JA biosynthesis and signaling and are involved in regulating the JA responses to –P_i_, alkalinity, salinity, cold, and other stresses (Aparicio-Fabre et al. 2013; Zhu et al. 2014; Goossens et al. 2016). Finally, AT4G35160 (*Glyma.10G176500*, identified in roots of both –Fe and –P_i_ roots) is involved in melatonin synthesis. The application of melatonin promotes the development of lateral and adventitious roots and, through free radical scavenging, protects plants from a variety of abiotic stresses. Plants with enhanced melatonin may be better equipped to survive abiotic and biotic stress. The identification of genes conserved between *Arabidopsis* and soybean and between dehydration and nutrient deficiency stress lends additional support to the classification of these genes as memory genes.

Genes unique to the T1T2 timepoint for both –Fe- and –P_i_-stressed plants represent a novel type of memory gene classification. In plants exposed to a single stress event (T2), these genes were not differentially expressed, suggesting that the differential expression of these genes requires prior stress exposure. In our data, we identified 247 and 3128 genes from leaves and roots, respectively, expressed only at the T1T2 timepoint (Online Resource 13). Gene Ontology analyses of these genes in leaves revealed they are involved in response to heat (GO:0009408, 17 genes) and nitrate transport (GO:0015706, 13 genes). In roots, significantly overrepresented GO processes included protein targeting to the mitochondria (GO:0006626, 47 genes), pyrimidine biosynthesis (GO:0009220, 51 genes), protein import to the nucleus (GO:0006606, 36 genes), pollen tube reception (GO:0010483, 10 genes), and RNA methylation (GO:0001510, 50 genes). The 47 genes associated with protein import to mitochondria are important to repair damaged organelles and to increase the size of mitochondria during regrowth. These include four genes encoding heat shock 60 proteins (*Glyma.10G127800*, *Glyma.10G193200*, *Glyma.20G079300*, and *Glyma.20G197100*) which are involved in folding mitochondrial pre-proteins, an essential step in mitochondrial protein repair and biogenesis (Voos 2013). The mitochondria are known to integrate signals from stress response pathways, translating stress signals into energy deficiency signals and shifting nuclear gene expression to re-establishing metabolic balance (Jacoby et al. 2011; Liberatore et al. 2016). Genes involved in these processes include *Glyma.07G152200* and *Glyma.12G154400*, which encode important components of the electron transport chain and cytochrome c oxidase, respectively. Previous studies have shown stress-tolerant cultivars exhibit enhanced expression of mitochondrial localized antioxidant defense genes (Liberatore et al. 2016). In *Arabidopsis*, over 20% of stress-responsive proteins are targeted to the mitochondria (Taylor et al. 2009). Accordingly, genes encoding subunits involved in transporting proteins through the outer and inner mitochondrial membranes (*Glyma.06G166600*, *Glyma.08G367400*, *Glyma.09G002800*, *Glyma.10G040200*, *Glyma.12G06300*, *Glyma.13G000600*, and *Glyma.14G014300*) were identified as differentially expressed only at T1T2. This data re-affirms the importance of mitochondrial activity in the nutrient stress response in plants and is the first recognition of this response in soybean. We also identified a number of genes involved in post-transcriptional gene silencing and methylation. These include XRN3 (*Glyma.12G093500*), which is a post-transcriptional gene silencing suppressor (Gy et al. 2007), and AT5G26180 (*Glyma.04G054300*), which methylates nucleosides in response to stress (Wang et al. 2017). The identification of genes associated with methylation uniquely differentially expressed at T1T2 suggests plants may utilize changes in methylation or post-transcriptional gene silencing as part of their stress response as proposed by recent studies (Crisp et al. 2016). Further, these responses may differ in response to repeated stresses compared to an initial stress exposure. Further research will be required to tease out the roles of these processes in the soybean stress response.

### Leveraging QTL mapping studies and gene expression data

The QTL on soybean chromosome 3 accounts for over 70% of the phenotypic response to –Fe conditions and has been identified in multiple studies (Diers et al. 1992; Lin et al. 2000; Peiffer et al. 2012; Mamidi et al. 2014). However, identifying the candidate gene(s) within the QTL region has proven difficult. Severin et al. (2010) narrowed the iron inefficiency introgression to a 4.2-Mb region using RNA-seq analyses. Peiffer et al. (2012) further narrowed this region and hypothesized a deletion within the dimerization domain of bHLH038 homologs (*Glyma.03G130400* and *Glyma.03G130600*) which resulted in susceptibility to iron stress. While the expression of these transcription factors is induced by iron stress, no follow-up experiments have confirmed the role of these genes in the soybean iron deficiency response. By examining all the differentially expressed genes identified in this study that map within this QTL, we noted several genes that could play a role in iron deficiency responses. Immediately downstream of the bHLH038 homologs is a gene (*Glyma.03G130700*) highly differentially expressed in response to iron stress (Online Resource 16a). Unfortunately, no annotation information exists for this gene because of its small size. We also identified a cluster of four genes (*Glyma.03G162400*–*Glyma.03G162700*), which are all members of the ERF TF family (two ERF98 homologs and two ERF15 homologs) and all highly downregulated at T1T2 and T2 in response to both –Fe and –P_i_ stress. In *Arabidopsis*, ERF98 regulates ascorbic acid biosynthesis (Zhang et al. 2012), which mitigates reactive oxygen species produced by abiotic stress. Similarly, ERF15 positively regulates ABA and confers immunity against *P. syringae* (Lee et al. 2014). Both ascorbic acid and ABA play key roles in defense and development processes, both of which are affected by –Fe. These genes may play important roles in translating and regulating the iron deficiency response.

Since soybean has undergone multiple genome duplication events, we also examined the region homeologous to the chromosome 3 QTL. This region on chromosome 19 contains simple sequence repeat marker Satt481 which was associated with IDC resistance by Charlson et al. (2005), confirming this region is also important for IDC tolerance. Assuming that a gene shared between these regions was responsible for IDC tolerance, we plotted the expression of all the Gm19 genes that had a homeolog on the chromosome 3 IDC QTL (Online Resource 16b). Again, the small, unannotated gene immediately downstream of the bHLH038 TFs stands out as do a cluster of four ERF transcription factors near the bottom of the QTL, homeologs of the *Glyma.03162400*–*Glyma.03G162700* cluster. We hypothesize the genes conserved between the two regions and those exhibit dynamic expression changes in response to –Fe stress are novel high-target IDC tolerance candidate genes. Future work by our group will focus on elucidating the role of these genes in the soybean nutrient stress response.

## Conclusion

This study allowed us to directly compare gene expression profiles of plants exposed to –Fe and –P_i_ simultaneously at multiple timepoints. These comparisons confirm that soybean utilizes the same genes and biological pathways in response to both micro- and macronutrient deficiencies. However, our data also clearly demonstrates that while soybean quickly responses to changes in nutrient deficiencies (within 24 h), soybean responded to –Fe deficiency, but P_i_ resupply. We hypothesize that the conserved responses observed between –Fe- and –P_i_-stressed roots result from both shared molecular pathways used by both nutrients and the ability of roots to quickly respond to altered nutrient availability and achieve a new homeostatic state within the 24-h period. Conversely, the differences observed between –Fe- and –P_i_-stressed leaves illustrates leaves respond slower and induce multiple responses to the new nutrient status. We also determined that soybean utilizes stress priming mechanisms, which may include increasing mitochondrial antioxidant defense gene expression and the utilization of methylation and gene silencing, to alter gene expression profiles in response to repeated stress exposures. Integrating the results of individual analyses allowed us to identify unique TFs from multiple TFFs that interact to regulate specific biological processes. This innovative approach can be applied to any RNA-seq analysis and should improve the biological utility of these datasets. Finally, combining previously identified QTL and differential gene expression patterns helped us identify genetic underpinnings of nutrient uptake and utilization, important qualities for improving and preserving crop yield. Together, this suite of data and analyses provides important biological insights into abiotic stress tolerance in crops and novel strategies to improve plant fitness and preserve yield.

## Electronic supplementary material

Online Resource 1 Clark genotype physiological response to -P_i_ stress. As concentration of available P_i_ is reduced in the hydroponic solutions, plants develop longer primary root with more lateral roots (a) while leaves of plants (b) have higher chlorophyll concentrations, making them appear a darker green. (PNG 2400 kb)

High Resolution Image (TIFF 1969 kb)

Online Resource 2 Multidimensional scaling analysis of leaf (A) and root (B) RNAseq samples. Samples names reflect original BAM files and indicate which samples were used. Nutrient stress is designated by either Pi (phosphate) or Fe (iron). Samples labeled Primary represent T1, Rec represents T1Rec, Mem represents T1T2, and Late represents T2. The letter preceding the final u indicates which plant was used, and the u designation represents the gene expression is being measured using only uniquely mapped reads. In panel A, the L designation represents leaf samples while in panel B the R designation represents root samples. (PNG 857 kb)

High Resolution Image (TIFF 2091 kb)

Online Resource 3 Pairwise comparisons of gene expression patterns of all root samples used in final RNAseq analyses. (PNG 1539 kb)

High Resolution Image (TIFF 2091 kb)

Online Resource 4 Pairwise comparisons of gene expression patterns of all leaf samples used in final RNAseq analyses. (PNG 1526 kb)

High Resolution Image (TIFF 2091 kb)

Online Resource 5 DEGs identified in –Fe and -P_i_ leaf samples (11,552 genes) with heatmap order information corresponding to Fig. 3 (XLSX 3172 kb)

Online Resource 6 DEGs identified in –Fe and -P_i_ root samples (19,357 genes) with heatmap order information corresponding to Fig. 3 (XLSX 5407 kb)

Online Resource 7 qRT-PCR analyses of 16 genes in 70 different tissue, nutrient stress, and stress timing combinations. Results of qRT-PCR confirm RNAseq expression measurements over 90% of the time. (XLSX 14 kb)

Online Resource 8 MA scatter-plot of gene expression patterns measured by RNAseq compared to gene expression measured by qRT-PCR. *R*^2^ reflects positive correlation between the two methods, with RNAseq displaying increased expression compared to qRT-PCR 74% of the time. (JPG 48 kb)

Online Resource 9 Over-represented Biological Process Gene Ontology (GO) terms identified at each time-point for both leaves and roots of -Fe and -P_i_ stressed plants. (XLSX 67 kb)

Online Resource 10 Over-represented transcription factor families (TFFs) identified at each timepoint in leaves and roots of -Fe and -P_i_ stressed plants. (XLSX 11 kb)

Online Resource 11 DEGs identified as opposite memory genes in leaf and root samples from -Fe and -P_i_ stressed plants (789 genes) (XLSX 259 kb)

Online Resource 12 Gene expression patterns (as Z-scores) of genes expressed in the opposite direction after a second stress exposure compared to the first stress exposure (memory genes) identified in leaves (a) and roots (b). This analysis identified 333 genes in leaves and 486 genes in roots. Cluster analysis identified four unique clusters in leaves. Clusters one and four are associated with unique biological processes: genes in cluster one are associated with growth and development, vascular formation, and lignin catabolism while genes in cluster four are associated with signal transduction and the generation of energy and metabolites. In roots, 486 memory genes were identified and cluster analysis identified five unique clusters. Again, clusters are associated with unique biological processes: genes in cluster one are involved in heat responses, cluster two cell wall biosynthesis, and genes in cluster five are associated with defense processes. (PNG 387 kb)

High Resolution Image (TIFF 1969 kb)

Online Resource 13 DEGs identified as T1T2 unique memory genes in leaf and root samples from -Fe and -P_i_ stressed plants (1943 genes total). (XLSX 1278 kb)

Online Resource 14 Identification and expression patterns of high priority candidate genes within previously identified P_i_ QTLs. Previously identified P_i_ QTL regions on chromosomes 2, 8, and 18 (panels a, b, and c, respectively). All genes within the QTL region are denoted in the row titled genes. Those that are differentially expressed at any given treatment timepoint are denoted by colored circles in the row corresponding to the treatment in which they were found to be differentially expressed. All expression is provided as Log2 fold changes. Genes in yellow were up-regulated compared to control plants while genes in blue were downregulated compared to control plants. Chromosome 2 spans 115 genes and contains six high priority candidate genes including two phospholipases (A), and a single copy of a leucine rich receptor like kinase involved in hormone and abiotic stress signal transduction (ten Hove et al. 2011) (B), Ferritin (C), a gene with no known annotation (D), and ACO4 (E) a component of the ethylene biosynthesis pathway. The QTL on chromosome 8 spans 27 genes and yet contains six high priority candidate genes; three MPK6 homologs involved in transport (A) and three phosphatases (B). The QTL on chromosome 18 spans 79 genes and contains eight high priority candidate genes including an SPX homolog (A), a heat shock protein (B), four receptor like proteins (C), a highly differentially expressed gene with no known annotations (D), and a homolog of ERF48 which imparts tolerance to multiple abiotic stresses (E). (PNG 1247 kb)

High Resolution Image (TIFF 1969 kb)

Online Resource 15 Transcription factor interactions and GO cluster associations. Arabidopsis homologs for each soybean transcription factor (TF) was identified and used for interaction analyses using Stringdb.org. Each circle represents a unique Arabidopsis TF corresponding to a DE soybean TF. Lines between TFs illustrate interactions. Combining this data with the GO clustering information by coloring TFs associated with a single GO cluster using the colors assigned to clusters in Fig. 4, identifies groups of TFs from multiple TFFs interacting to regulate unique biological processes. TFs associated with multiple GO clusters are colored in grey. The DE TFs from leaves (a) are highly interconnected with distinct regions associated with individual GO clusters. The interaction network of DE TFs from roots (b) is more distinct, but the GO clusters are highly interconnected except in rare instances where a single GO cluster is highly represented. (PNG 705 kb)

High Resolution Image (TIFF 1969 kb)

Online Resource 16 Differential expression of genes conserved between Fe QTL. Differential gene expression in genes conserved between the canonical Gm03 (panel a) iron QTL and homologous QTL region on chromosome Gm19 (panel b). The genes present in these QTL regions are depicted in the row labeled genes. Genes differentially expressed genes in response to –Fe or –P_i_ stress in roots (R) or leaves (L) are depicted in the rows corresponding to the timepoint where they were identified. Expression is provided as Log2 fold change. Yellow indicates increased expression compared to control plants and purple indicated reduced expression. The blue dashed line denotes the location of the two bHLH038 homologs identified as the putative candidate gene by (Peiffer et al. 2012). Genes highlighted by arrows represent high-interest candidate genes as identified by increased levels of differential expression in response to –Fe stress and conserved responses in the homologous region. Gene labeled A has no annotation while genes labeled B are both annotated as ERF transcription factors. (PNG 821 kb)

High Resolution Image (TIFF 1969 kb)
