# Supplementary figures and images for: Dynamic gene expression changes in response to micronutrient, macronutrient, and multiple stress exposures in soybean
Source: Funct Integr Genomics. 2019 Oct 26;20(3):321–41. doi: 10.1007/s10142-019-00709-9 (PMC7152590; doi:10.1007/s10142-019-00709-9)

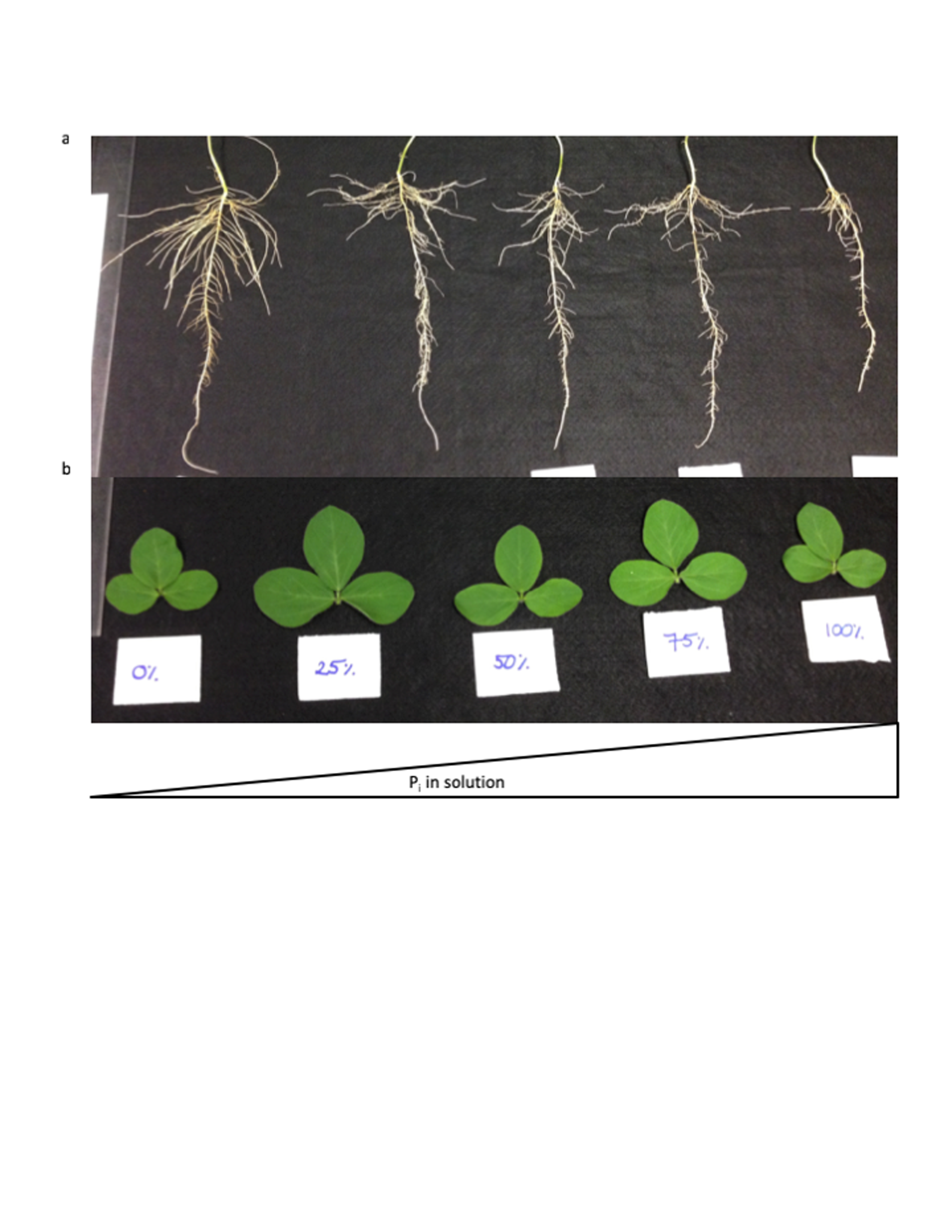

Supplement: Supplementary file 1 — Clark genotype physiological response to -Pi stress. As concentration of available Pi is reduced in the hydroponic solutions, plants develop longer primary root with more lateral roots (a) while leaves of plants (b) have higher chlorophyll concentrations, making them appear a darker green. (PNG 2400 kb) [file 10142_2019_709_Fig8_ESM.png]

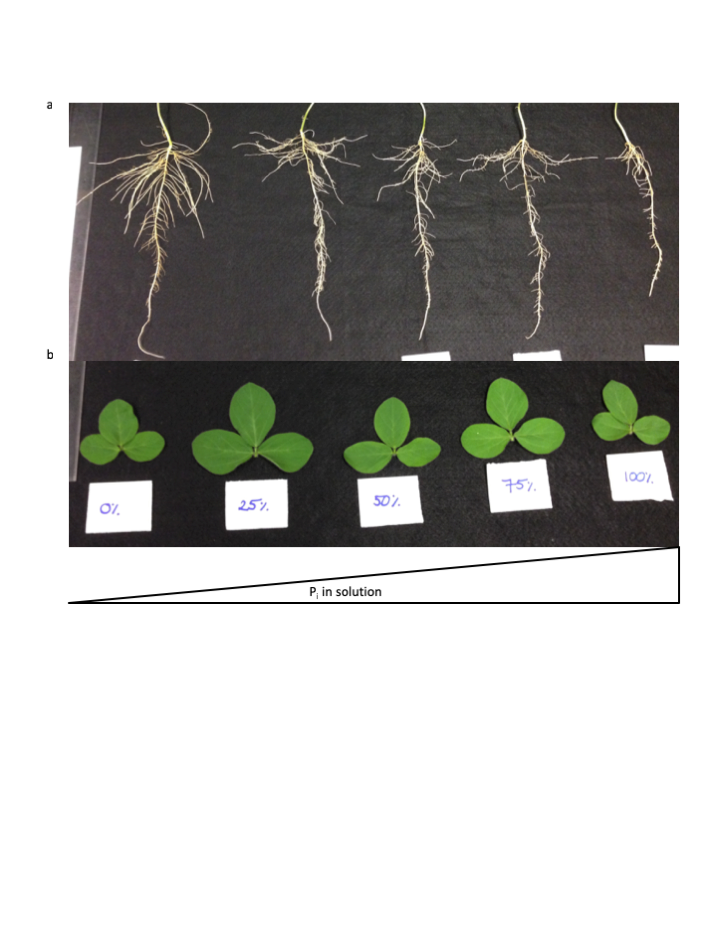

Supplement: Supplementary file 2 — High Resolution Image (TIFF 1969 kb) [file 10142_2019_709_MOESM1_ESM.tiff]

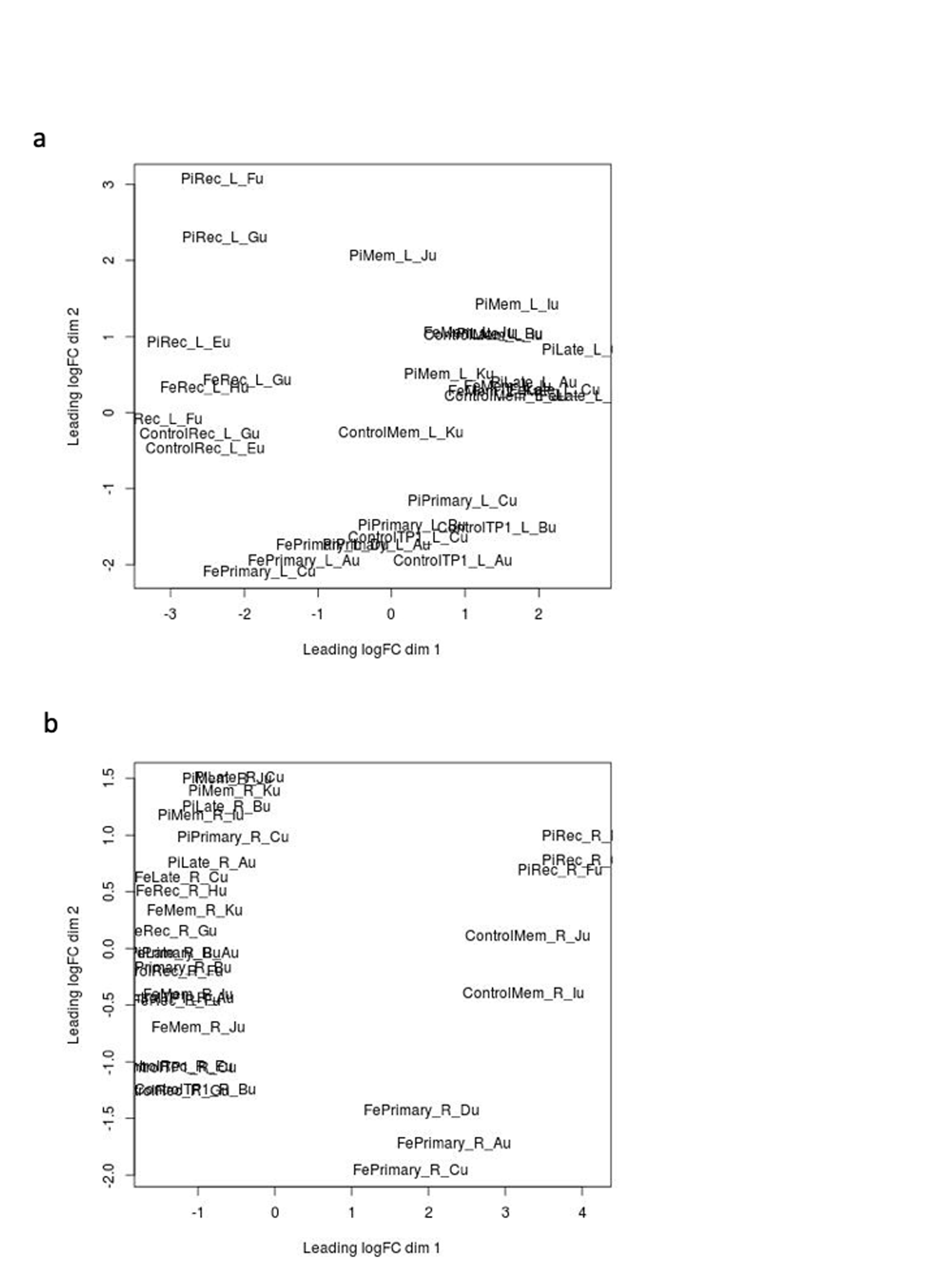

Supplement: Supplementary file 3 — Multidimensional scaling analysis of leaf (A) and root (B) RNAseq samples. Samples names reflect original BAM files and indicate which samples were used. Nutrient stress is designated by either Pi (phosphate) or Fe (iron). Samples labeled Primary represent T1, Rec represents T1Rec, Mem represents T1T2, and Late represents T2. The letter preceding the final u indicates which plant was used, and the u designation represents the gene expression is being measured using only uniquely mapped reads. In panel A, the L designation represents leaf samples while in panel B the R designation represents root samples. (PNG 857 kb) [file 10142_2019_709_Fig9_ESM.png]

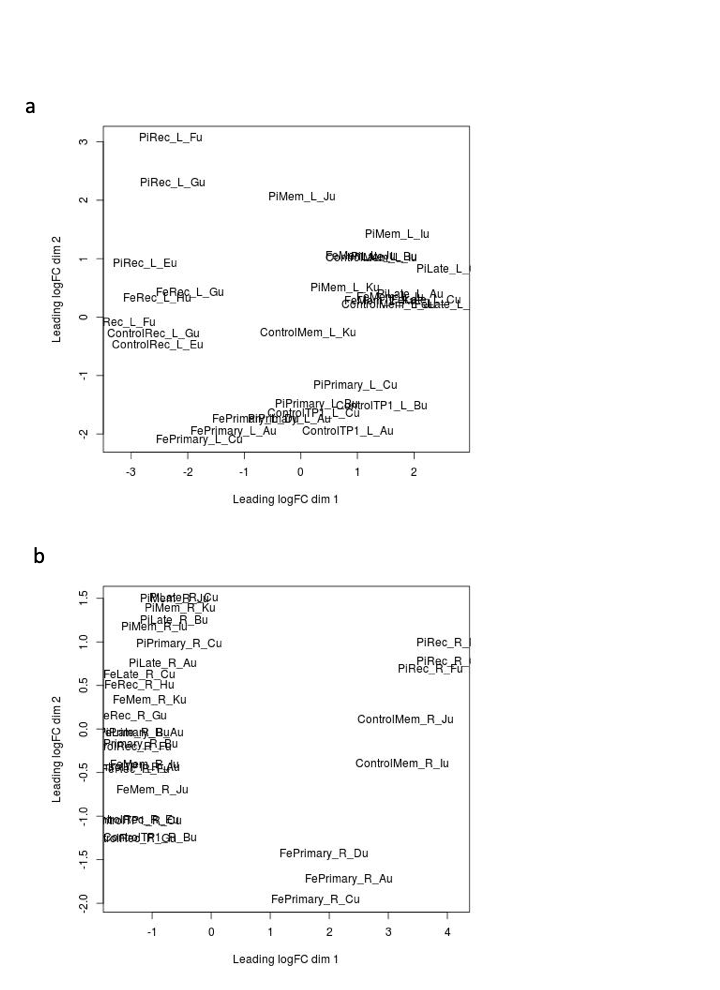

Supplement: Supplementary file 4 — High Resolution Image (TIFF 2091 kb) [file 10142_2019_709_MOESM2_ESM.tiff]

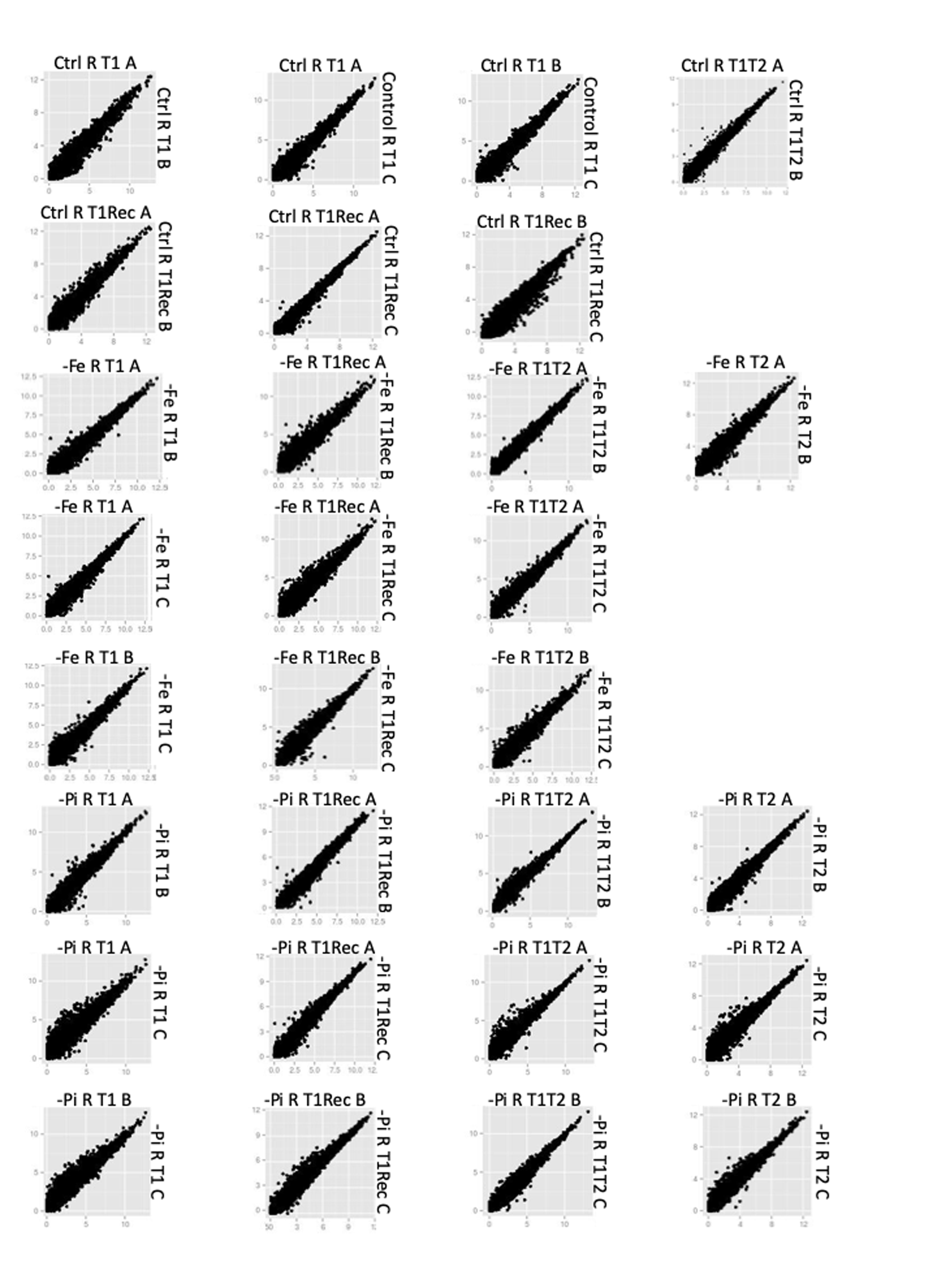

Supplement: Supplementary file 5 — Pairwise comparisons of gene expression patterns of all root samples used in final RNAseq analyses. (PNG 1539 kb) [file 10142_2019_709_Fig10_ESM.png]

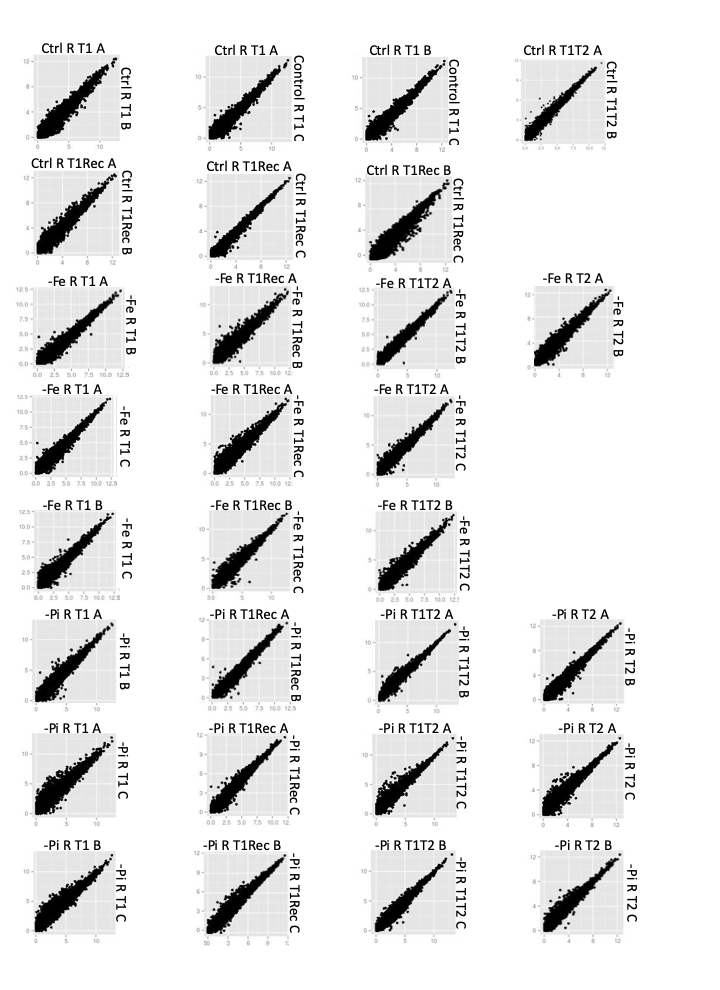

Supplement: Supplementary file 6 — High Resolution Image (TIFF 2091 kb) [file 10142_2019_709_MOESM3_ESM.tiff]

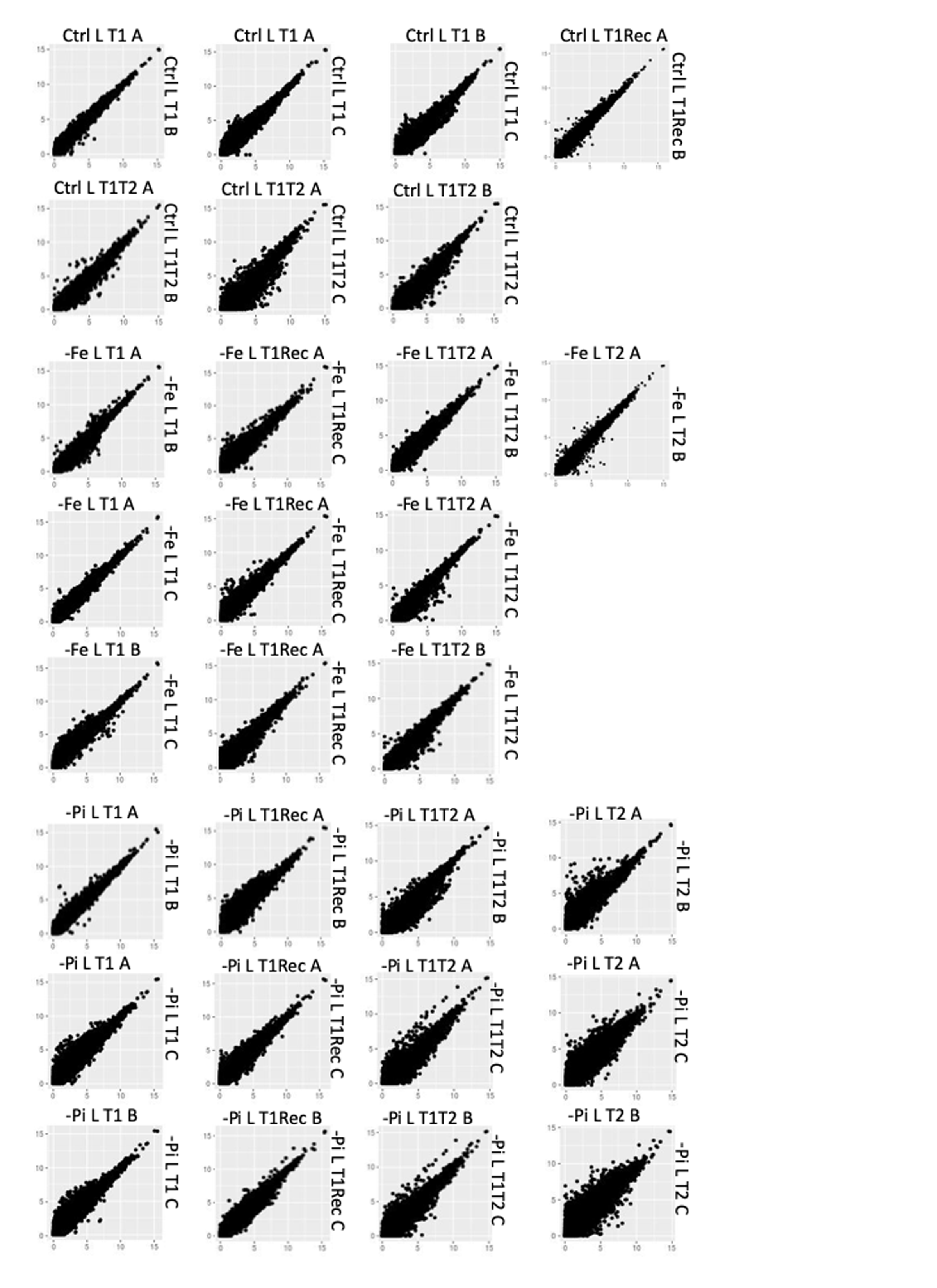

Supplement: Supplementary file 7 — Pairwise comparisons of gene expression patterns of all leaf samples used in final RNAseq analyses. (PNG 1526 kb) [file 10142_2019_709_Fig11_ESM.png]

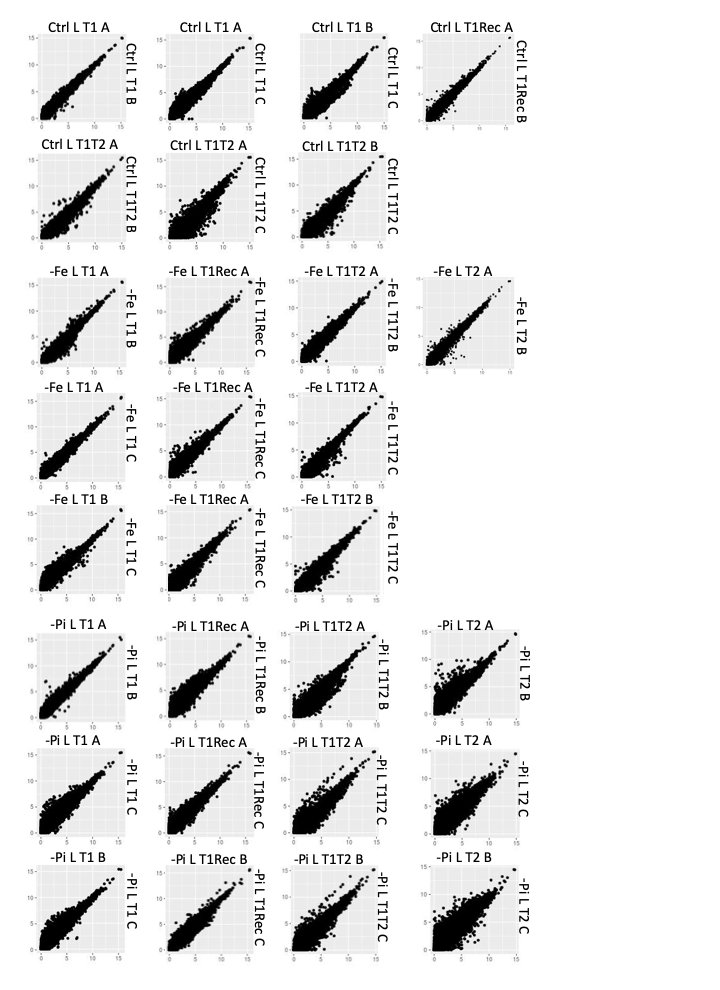

Supplement: Supplementary file 8 — High Resolution Image (TIFF 2091 kb) [file 10142_2019_709_MOESM4_ESM.tiff]

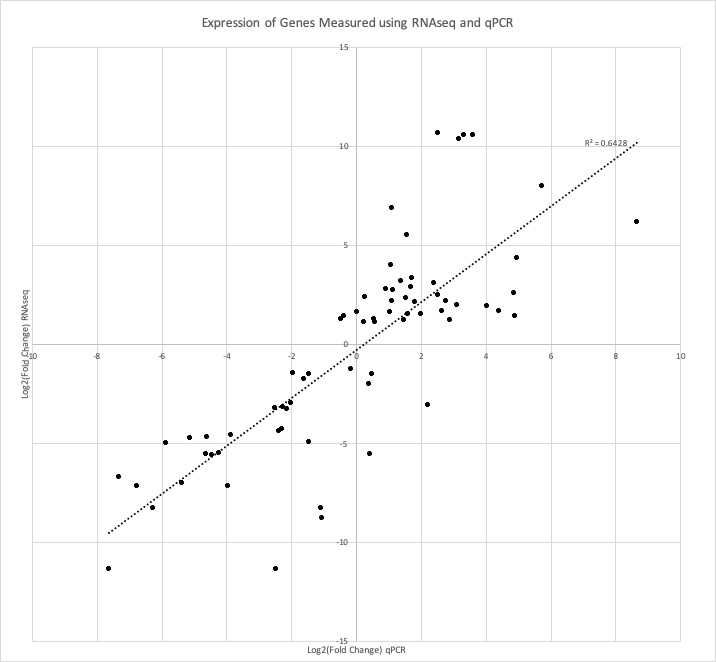

Supplement: Supplementary file 12 — MA scatter-plot of gene expression patterns measured by RNAseq compared to gene expression measured by qRT-PCR. R2 reflects positive correlation between the two methods, with RNAseq displaying increased expression compared to qRT-PCR 74% of the time. (JPG 48 kb) [file 10142_2019_709_MOESM8_ESM.jpg]

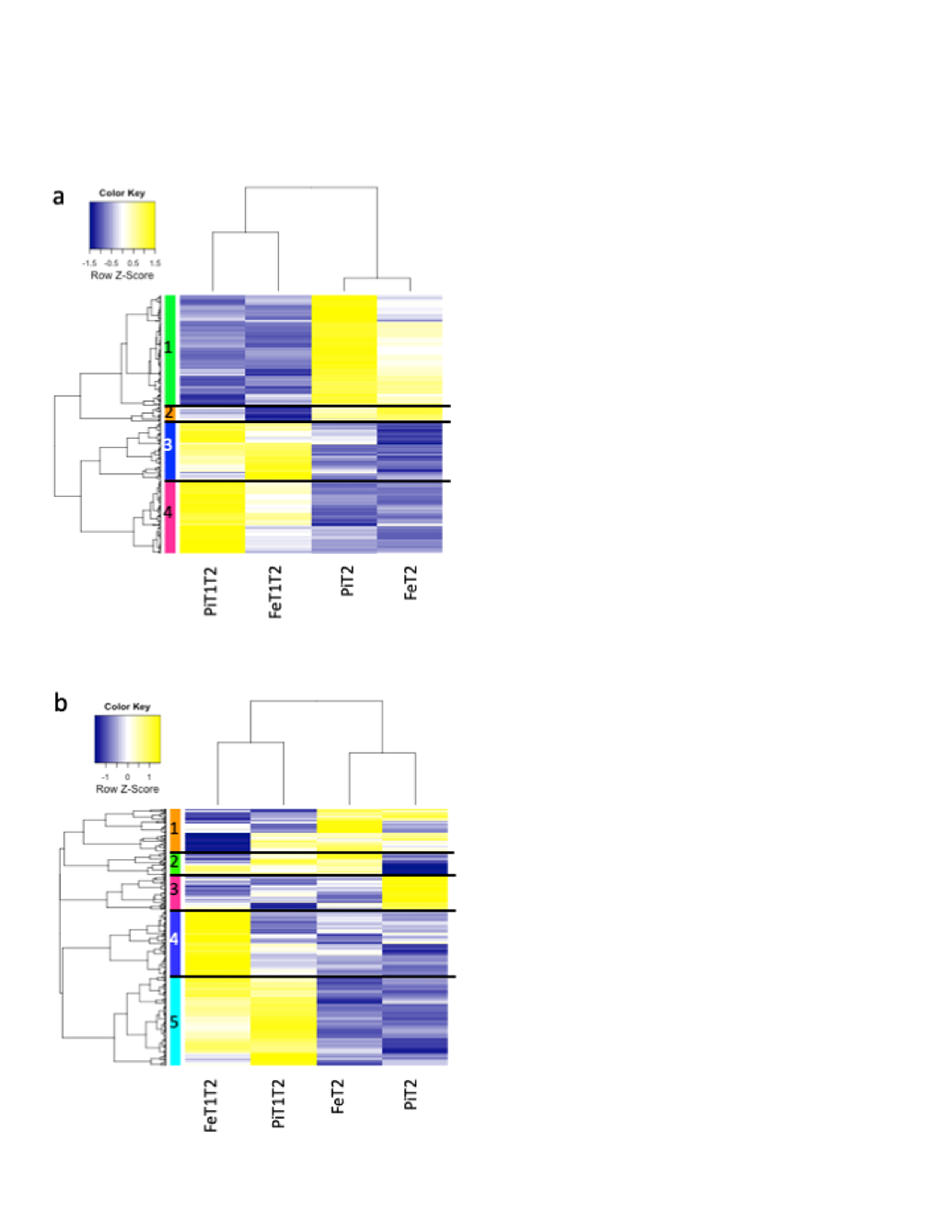

Supplement: Supplementary file 16 — Gene expression patterns (as Z-scores) of genes expressed in the opposite direction after a second stress exposure compared to the first stress exposure (memory genes) identified in leaves (a) and roots (b). This analysis identified 333 genes in leaves and 486 genes in roots. Cluster analysis identified four unique clusters in leaves. Clusters one and four are associated with unique biological processes: genes in cluster one are associated with growth and development, vascular formation, and lignin catabolism while genes in cluster four are associated with signal transduction and the generation of energy and metabolites. In roots, 486 memory genes were identified and cluster analysis identified five unique clusters. Again, clusters are associated with unique biological processes: genes in cluster one are involved in heat responses, cluster two cell wall biosynthesis, and genes in cluster five are associated with defense processes. (PNG 387 kb) [file 10142_2019_709_Fig12_ESM.png]

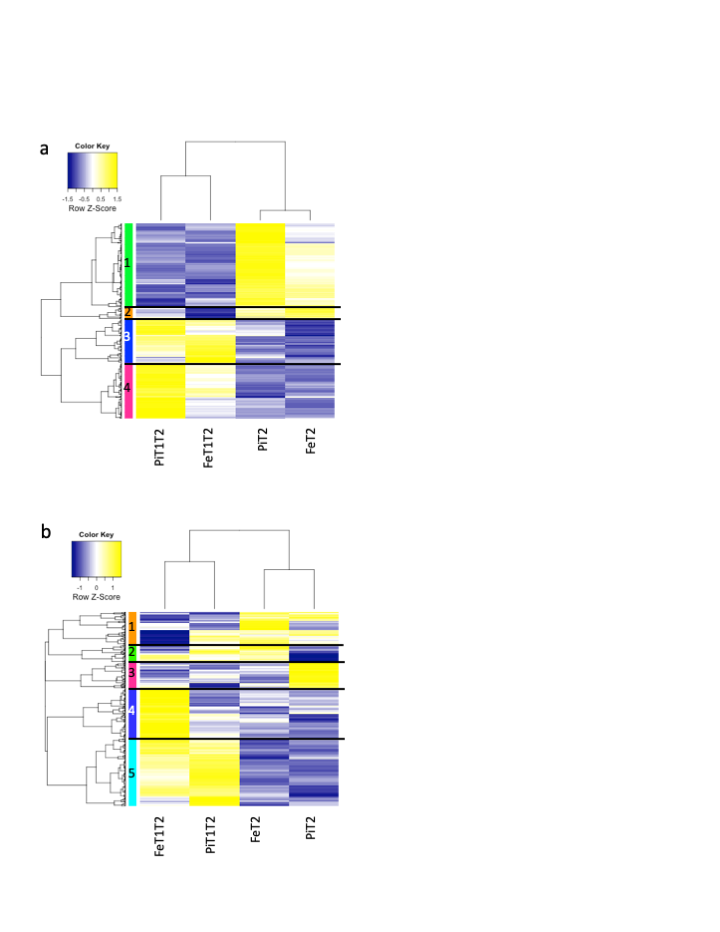

Supplement: Supplementary file 17 — High Resolution Image (TIFF 1969 kb) [file 10142_2019_709_MOESM12_ESM.tiff]

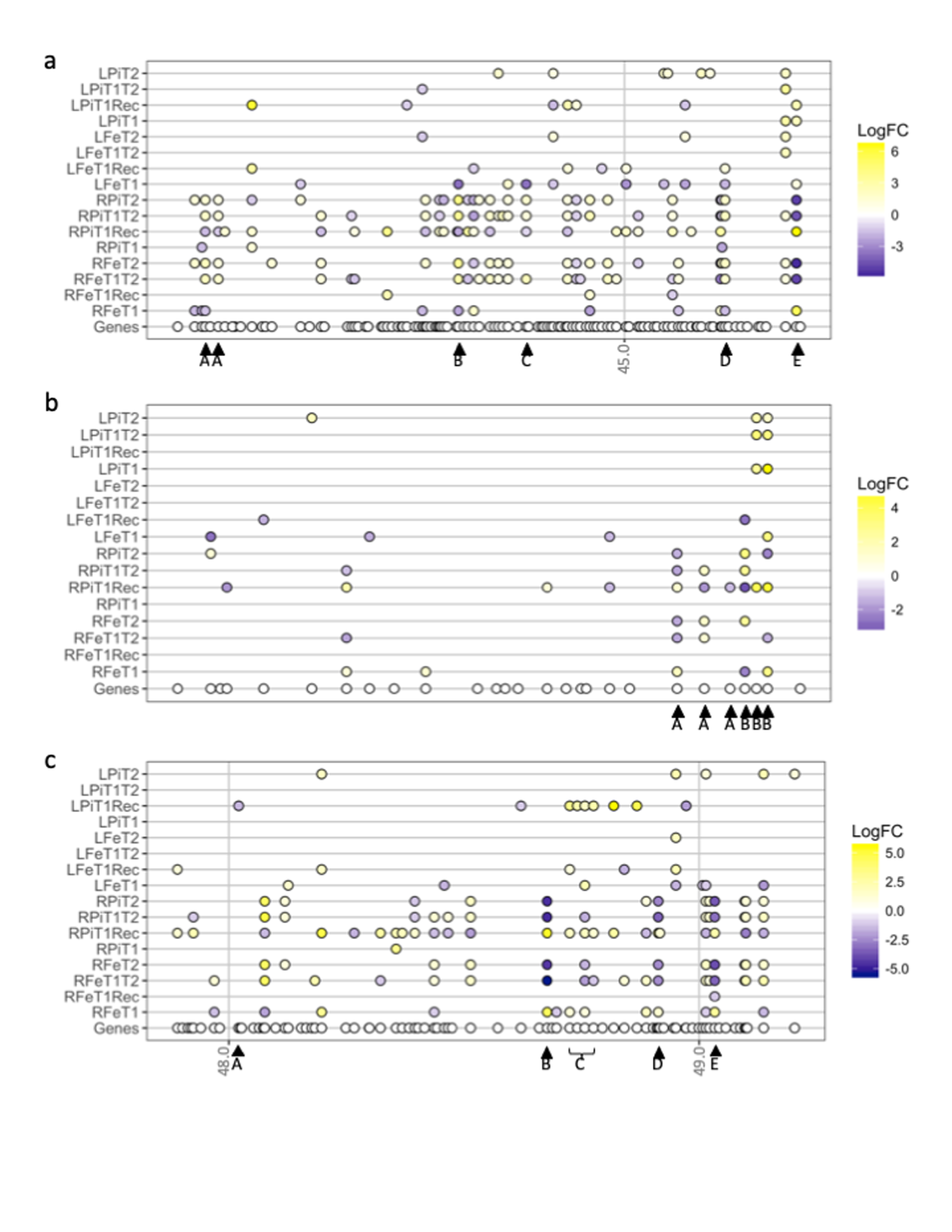

Supplement: Supplementary file 19 — Identification and expression patterns of high priority candidate genes within previously identified Pi QTLs. Previously identified Pi QTL regions on chromosomes 2, 8, and 18 (panels a, b, and c, respectively). All genes within the QTL region are denoted in the row titled genes. Those that are differentially expressed at any given treatment timepoint are denoted by colored circles in the row corresponding to the treatment in which they were found to be differentially expressed. All expression is provided as Log2 fold changes. Genes in yellow were up-regulated compared to control plants while genes in blue were downregulated compared to control plants. Chromosome 2 spans 115 genes and contains six high priority candidate genes including two phospholipases (A), and a single copy of a leucine rich receptor like kinase involved in hormone and abiotic stress signal transduction (ten Hove et al. 2011) (B), Ferritin (C), a gene with no known annotation (D), and ACO4 (E) a component of the ethylene biosynthesis pathway. The QTL on chromosome 8 spans 27 genes and yet contains six high priority candidate genes; three MPK6 homologs involved in transport (A) and three phosphatases (B). The QTL on chromosome 18 spans 79 genes and contains eight high priority candidate genes including an SPX homolog (A), a heat shock protein (B), four receptor like proteins (C), a highly differentially expressed gene with no known annotations (D), and a homolog of ERF48 which imparts tolerance to multiple abiotic stresses (E). (PNG 1247 kb) [file 10142_2019_709_Fig13_ESM.png]

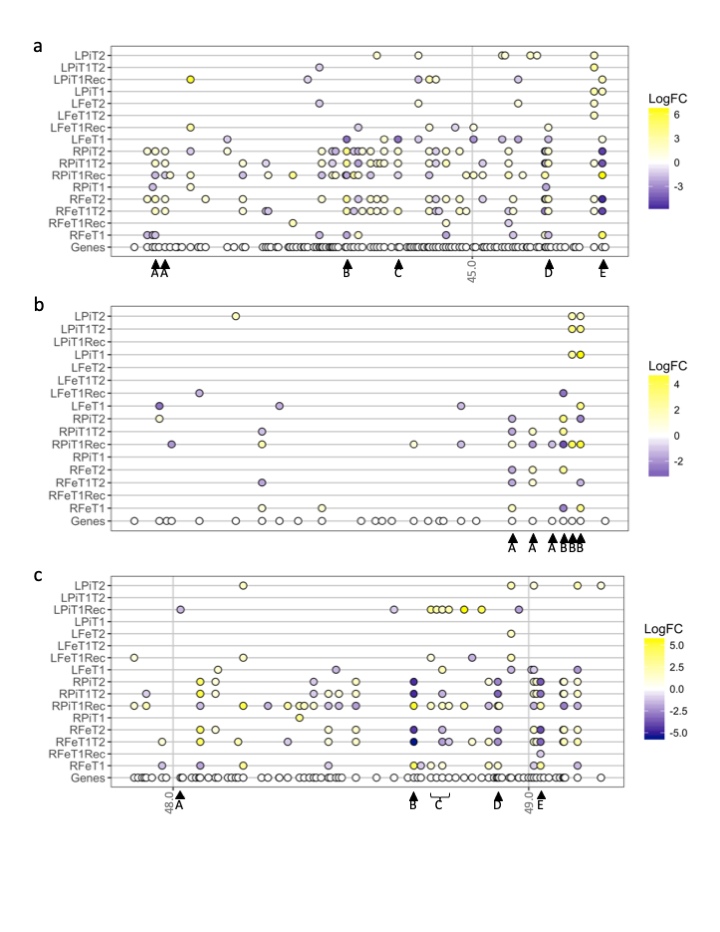

Supplement: Supplementary file 20 — High Resolution Image (TIFF 1969 kb) [file 10142_2019_709_MOESM14_ESM.tiff]

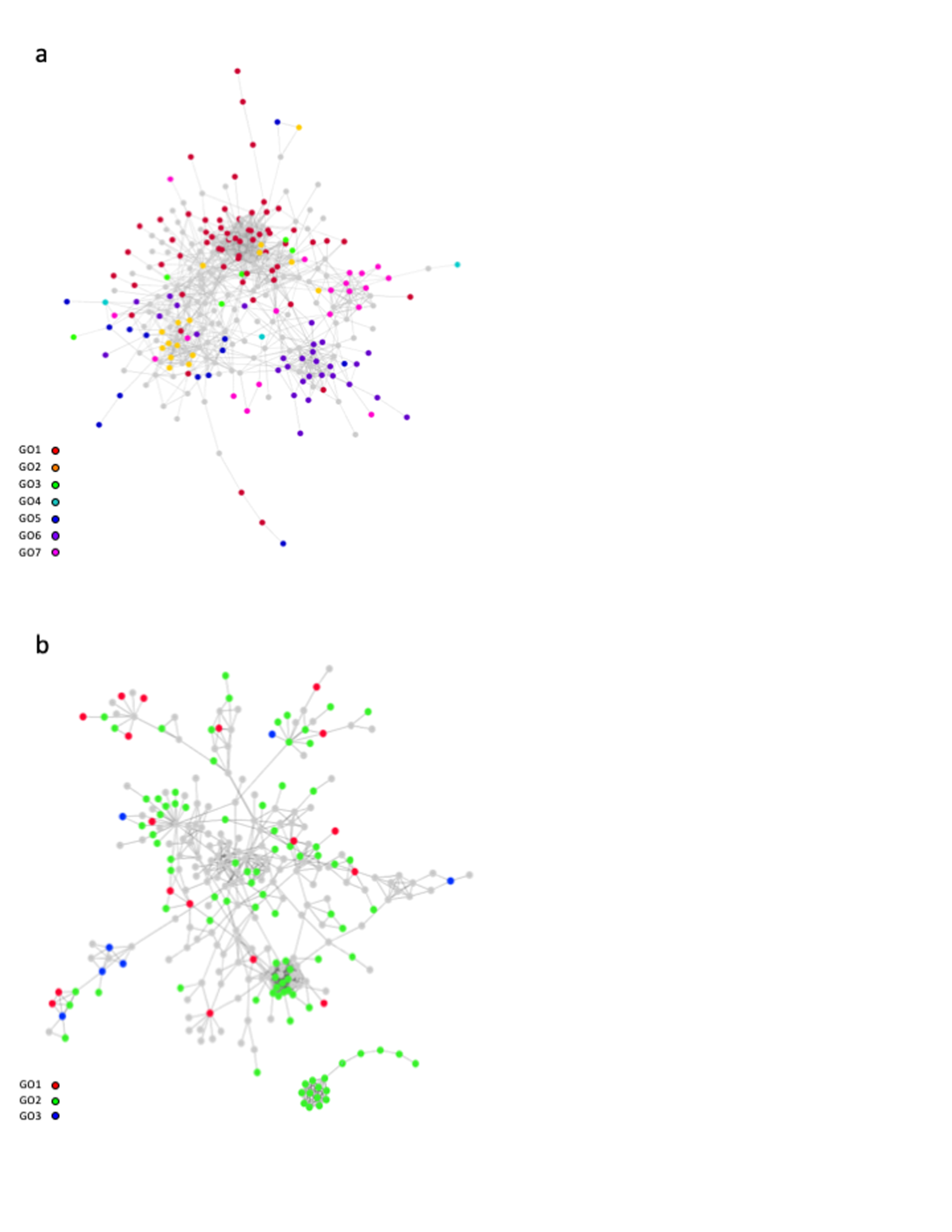

Supplement: Supplementary file 21 — Transcription factor interactions and GO cluster associations. Arabidopsis homologs for each soybean transcription factor (TF) was identified and used for interaction analyses using Stringdb.org. Each circle represents a unique Arabidopsis TF corresponding to a DE soybean TF. Lines between TFs illustrate interactions. Combining this data with the GO clustering information by coloring TFs associated with a single GO cluster using the colors assigned to clusters in Fig. 4, identifies groups of TFs from multiple TFFs interacting to regulate unique biological processes. TFs associated with multiple GO clusters are colored in grey. The DE TFs from leaves (a) are highly interconnected with distinct regions associated with individual GO clusters. The interaction network of DE TFs from roots (b) is more distinct, but the GO clusters are highly interconnected except in rare instances where a single GO cluster is highly represented. (PNG 705 kb) [file 10142_2019_709_Fig14_ESM.png]

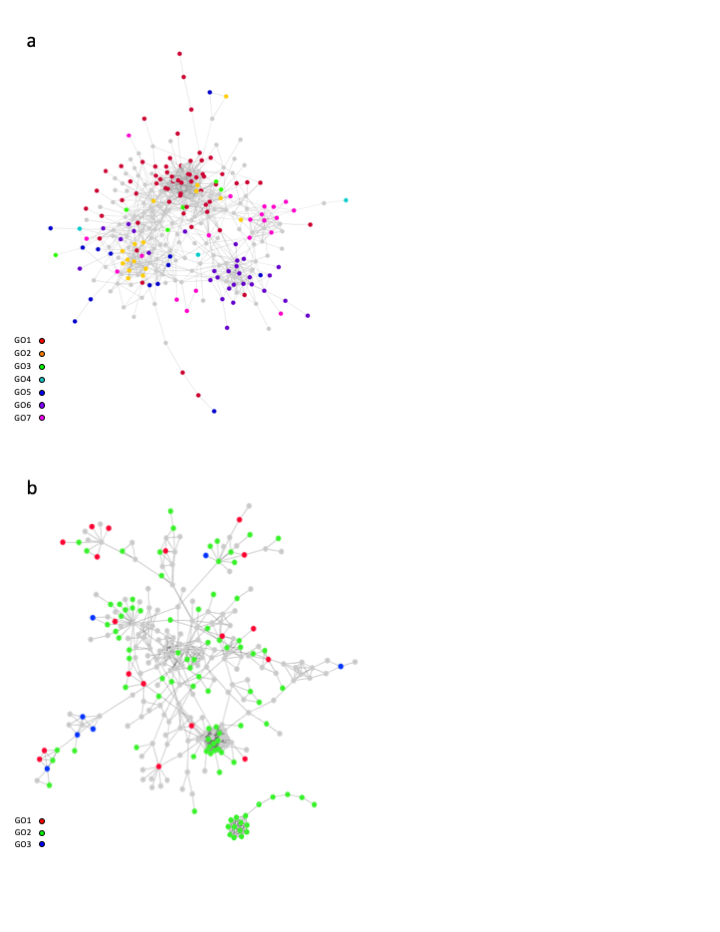

Supplement: Supplementary file 22 — High Resolution Image (TIFF 1969 kb) [file 10142_2019_709_MOESM15_ESM.tiff]

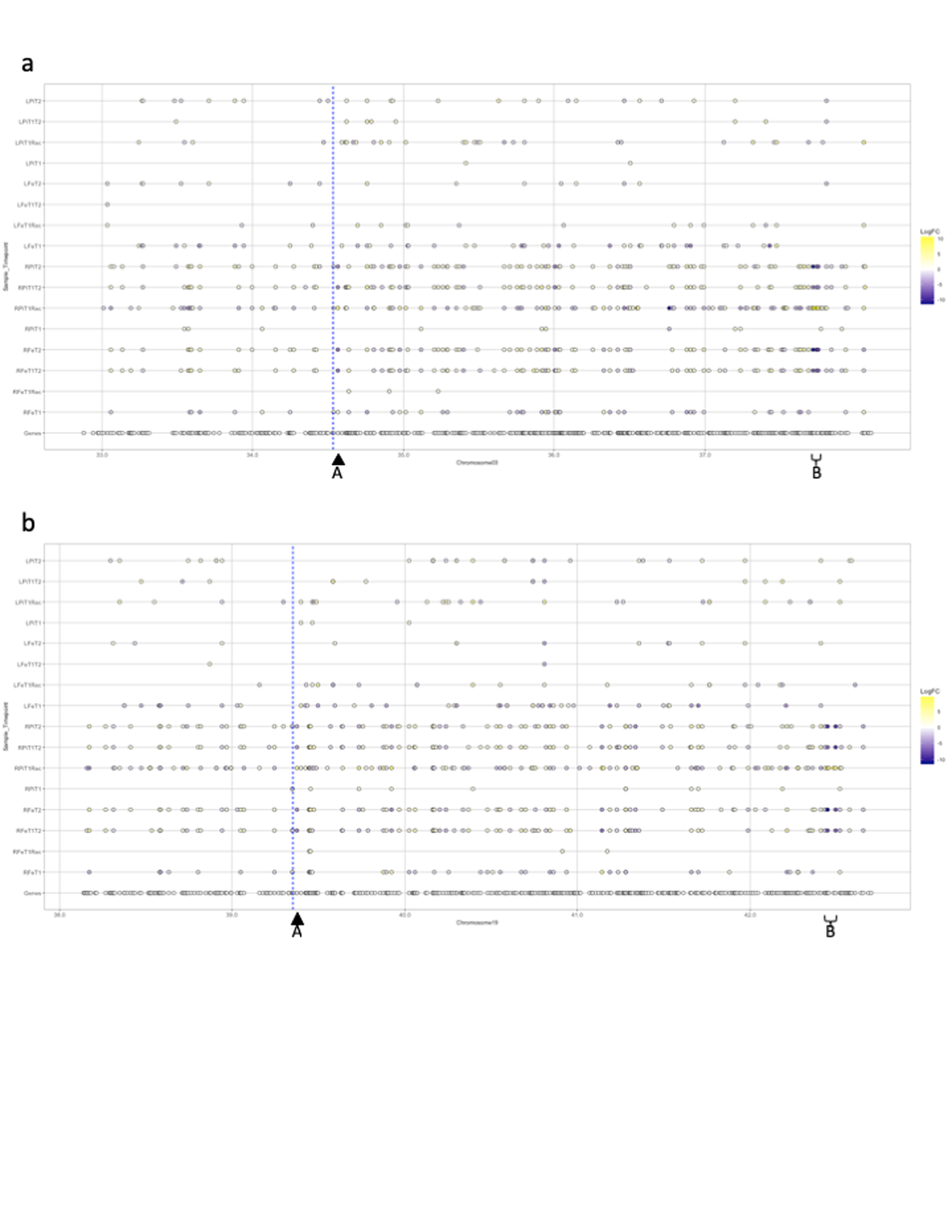

Supplement: Supplementary file 23 — Differential expression of genes conserved between Fe QTL. Differential gene expression in genes conserved between the canonical Gm03 (panel a) iron QTL and homologous QTL region on chromosome Gm19 (panel b). The genes present in these QTL regions are depicted in the row labeled genes. Genes differentially expressed genes in response to –Fe or –Pi stress in roots (R) or leaves (L) are depicted in the rows corresponding to the timepoint where they were identified. Expression is provided as Log2 fold change. Yellow indicates increased expression compared to control plants and purple indicated reduced expression. The blue dashed line denotes the location of the two bHLH038 homologs identified as the putative candidate gene by (Peiffer et al. 2012). Genes highlighted by arrows represent high-interest candidate genes as identified by increased levels of differential expression in response to –Fe stress and conserved responses in the homologous region. Gene labeled A has no annotation while genes labeled B are both annotated as ERF transcription factors. (PNG 821 kb) [file 10142_2019_709_Fig15_ESM.png]

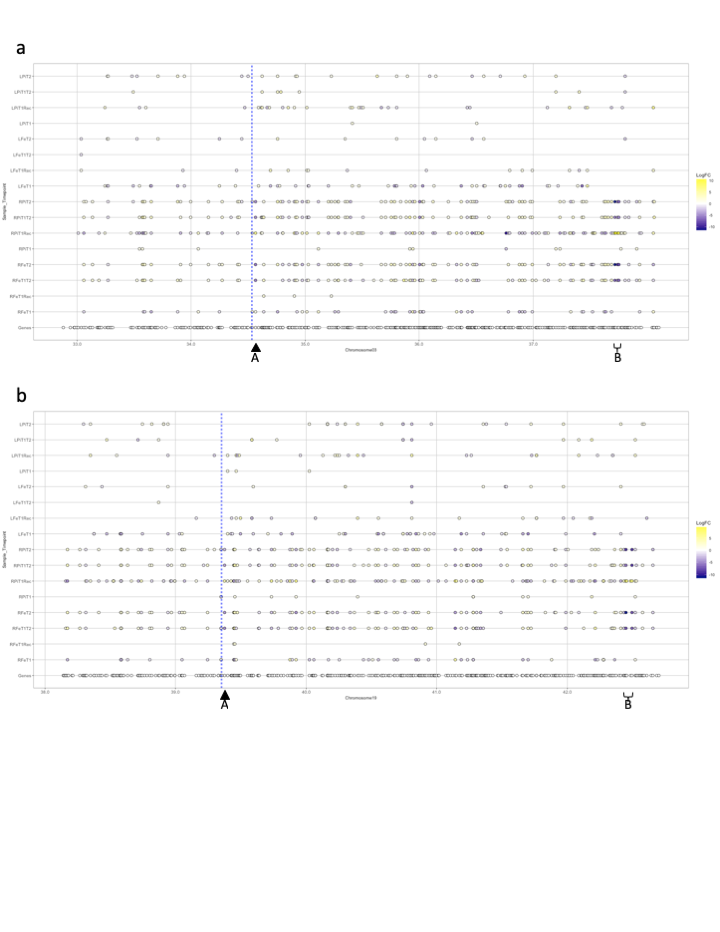

Supplement: Supplementary file 24 — High Resolution Image (TIFF 1969 kb) [file 10142_2019_709_MOESM16_ESM.tiff]
